# Structural and genome-wide analyses suggest that transposon-derived protein SETMAR alters transcription and splicing

**DOI:** 10.1016/j.jbc.2022.101894

**Published:** 2022-04-01

**Authors:** Qiujia Chen, Alison M. Bates, Jocelyne N. Hanquier, Edward Simpson, Douglas B. Rusch, Ram Podicheti, Yunlong Liu, Ronald C. Wek, Evan M. Cornett, Millie M. Georgiadis

**Affiliations:** 1 Department of Biochemistry and Molecular Biology, Indiana University School of Medicine, Indianapolis, Indiana, USA; 2 Stark Neurosciences Research Institute, Indiana University School of Medicine, Indianapolis, Indiana, USA; 3 Department of Medical and Molecular Genetics, Indiana University School of Medicine, Indianapolis, Indiana, USA; 4 Center for Genomics and Bioinformatics, Indiana University, Bloomington, Indiana, USA

**Keywords:** SETMAR, crystal structure, terminal inverted repeat, differential gene expression, alternative splicing, AS, alternative splicing, 5-BrdU, 5-bromodeoxyuridine, ChIP-Seq, chromatin immunoprecipitation sequencing, CMV4, cytomegalovirus 4, DBD, DNA-binding domain, DE, differentially expressed, FA, fluorescence anisotropy, FDR, false discovery rate, FL, full length, HEK293T, human embryonic kidney 293T cell line, HTH, helix–turn–helix, ILD, inclusion level difference, KMT, lysine methyltransferase, PDB, Protein Data Bank, PWM, position-weighted matrix, qPCR, quantitative PCR, SAD, single-wavelength anomalous diffraction, Se, selenium, SeMet, selenomethionine, sgRNA, single-guide RNA, snRNP, small nuclear ribonucleoprotein, TE, transposable element, TIR, terminal inverted repeat, TSS, transcription start site, UCSC, University of California Santa Cruz

## Abstract

Extensive portions of the human genome have unknown function, including those derived from transposable elements. One such element, the DNA transposon *Hsmar1*, entered the primate lineage approximately 50 million years ago leaving behind terminal inverted repeat (TIR) sequences and a single intact copy of the *Hsmar1* transposase, which retains its ancestral TIR-DNA-binding activity, and is fused with a lysine methyltransferase SET domain to constitute the chimeric *SETMAR* gene. Here, we provide a structural basis for recognition of TIRs by SETMAR and investigate the function of SETMAR through genome-wide approaches. As elucidated in our 2.37 Å crystal structure, SETMAR forms a dimeric complex with each DNA-binding domain bound specifically to TIR-DNA through the formation of 32 hydrogen bonds. We found that SETMAR recognizes primarily TIR sequences (∼5000 sites) within the human genome as assessed by chromatin immunoprecipitation sequencing analysis. In two SETMAR KO cell lines, we identified 163 shared differentially expressed genes and 233 shared alternative splicing events. Among these genes are several pre–mRNA-splicing factors, transcription factors, and genes associated with neuronal function, and one alternatively spliced primate-specific gene, *TMEM14B*, which has been identified as a marker for neocortex expansion associated with brain evolution. Taken together, our results suggest a model in which SETMAR impacts differential expression and alternative splicing of genes associated with transcription and neuronal function, potentially through both its TIR-specific DNA-binding and lysine methyltransferase activities, consistent with a role for SETMAR in simian primate development.

Although most of the transposable elements (TEs) that played essential roles in shaping modern eukaryotes are no longer active (1, 2), there is still much to discover about their legacy in shaping the function of the human genome. Almost half of the human genome is derived from TEs, primarily retrotransposons, whereas DNA transposons have contributed to about 3% of our genome (3). One DNA transposon, *Hsmar1*, was active in primates from about 50 to 37 million years ago (4) and gave rise to SETMAR (or Metnase), a fusion protein found only in simian (anthropoid) primates with an N-terminal SET domain and C-terminal *Hsmar1*-derived (MAR) transposase (5). MAR refers to the Hs mariner-derived domain. *SETMAR* encodes the only intact copy of the *Hsmar1* transposase in primates, although thousands of copies of its terminal inverted repeat (TIR) sequences, which flank the transposase gene in the ancestral transposon, remain. About two-thirds of the ∼7000 *Hsmar1* TIR-related sequences exist as single TIRs with the remainder in paired minielements (referred to as MITES or MADE1 elements) that can be up to 80 bp in length (4). These latter paired TIR sequences are analogous to nonautonomous elements described for other TEs and lack the transposase gene. In a recent report, MADE1 elements (80 bp) that contain two shortened 24 bp TIR elements (6, 7), many of which are variants of the consensus mariner-binding site (5), were reported as relevant SETMAR-binding sites based on analysis of chromatin immunoprecipitation sequencing (ChIP-Seq) data.

The search for a function for SETMAR in normal cells has proven challenging; this protein is only present in simian primates and cannot easily be studied in the context of an animal model lacking the TIR elements in its genome. SETMAR is expressed in most tissues with no distinguishing specificity within the brain or other tissues (8). To date, studies have relied on knockdown or overexpression studies to assess the function of SETMAR. There is general agreement on two activities associated with SETMAR. The first is retention of ancestral sequence-specific TIR-DNA-binding activity, mediated by the DNA-binding domain (DBD) of the transposase, although the ability to perform TIR-specific DNA cleavage events has been lost (5, 9, 10, 11). Ironically, none of the biological functions reported for SETMAR, nonhomologous end joining (12), chromosome decatenation (13), and restart of stalled replication forks (14), involve TIR-specific DNA-binding activity; the role of SETMAR in nonhomologous end joining remains controversial (15).

The second SETMAR function is lysine methyltransferase (KMT) activity, which is contained within the SET domain. There is, however, no consensus on the preferred substrate for the KMT activity. SETMAR was initially reported to dimethylate H3K36 (12) and was later reported to regulate gene expression through dimethylation of H3K36, a mark associated with open chromatin, mediated by sequence-specific DNA binding of SETMAR to intronic regions (6). However, a proteomics approach found no evidence that SETMAR methylates H3 within nucleosome substrates; SETMAR can weakly methylate isolated H3 *in vitro* but not on K36. Instead, SETMAR methylates K130 of a U1 splicing factor, small nuclear ribonucleoprotein (snRNP) 70 (16). The functional role of the KMT activity of SETMAR remains an open area of investigation.

In this study, we sought to identify a biological function for SETMAR–TIR interactions. To do this, we addressed the following key questions: How does SETMAR recognize TIR-DNA and which sequences represent preferred binding sites within the genome? What impact does SETMAR have on differential gene expression and does this role involve histone methylation? And perhaps most critically, what impact does SETMAR, a simian-specific protein, have on alternative splicing (AS), which is known to play a role in expanding the proteome in higher organisms (17). Here, we answer these questions and suggest a novel function for SETMAR in AS.

## Results and discussion

### SETMAR recognizes TIR-DNA through sequence-specific major groove and minor groove interactions

SETMAR comprises three structural domains, a SET domain, a transposase-derived DBD, and a catalytic domain. Of these, the DBD is highly conserved among the primate species in which this protein is expressed with only two variant amino acid residues (M332R and Q403H) (Fig. S1). Crystal structures of both the SET (Protein Data Bank [PDB] ID: 3BO5) and catalytic domains (PDB IDs: 3K9J and 3K9K) have been determined (18). To provide a structural basis for recognition of TIR-DNA by SETMAR, we used a selenomethionine (SeMet) phasing strategy (19) to determine the crystal structure of the SETMAR DBD comprising residues 329 to 440 complexed to a 25-mer DNA derived from the *Hsmar1* TIR at 2.37 Å resolution (Table S1 and Fig. S2). Although SETMAR DBD includes four intrinsic Met residues, three are located at the N terminus of this domain and were likely to be disordered. Thus as previously reported (19), a phasing strategy was devised in which strategic Met substitutions (I359M) (L423M) within two predicated alpha helical elements were introduced; these Met substitutions were critical for phasing. The C381R substitution was introduced to prevent disulfide bond formation.

**Figure 1:**
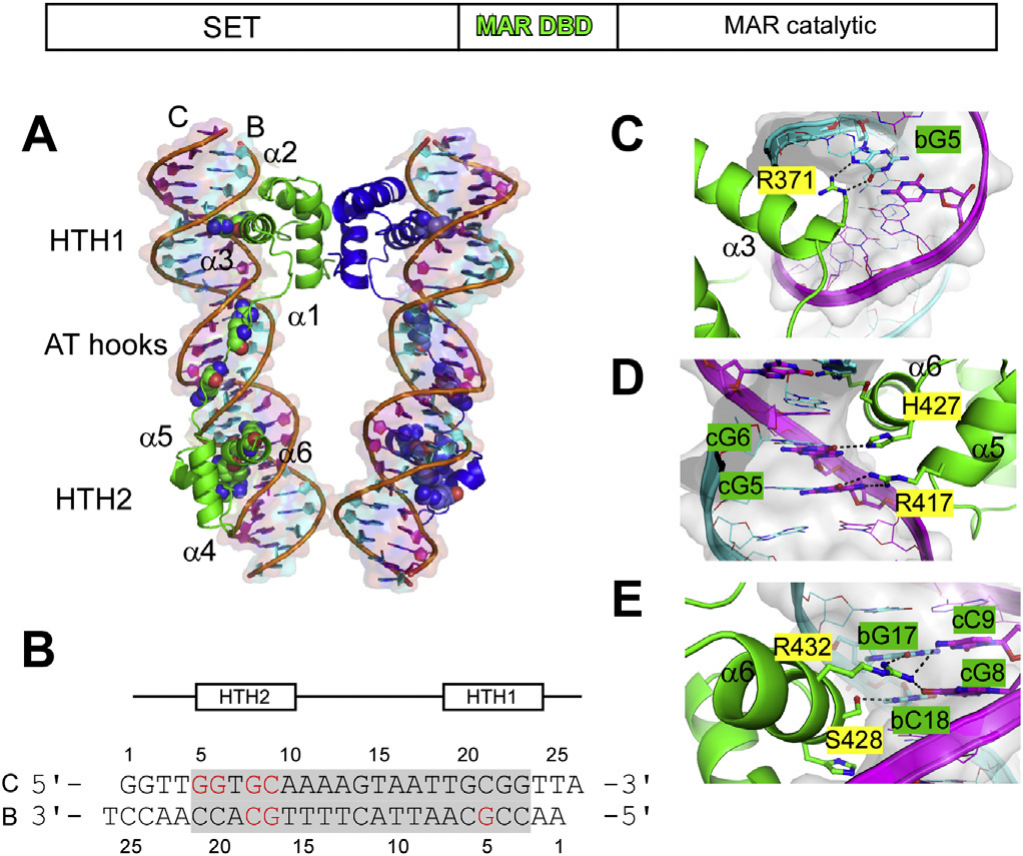
**Structural basis for SETMAR–TIR interactions.** A schematic of the SETMAR protein is shown with the SET domain and two domains derived from the mariner transposase gene MAR, DBD referring to the DBD derived from the *Hsmar1* transposase at the *top*. *A*, SETMAR recognizes TIR-DNA through interactions mediated by HTH1, HTH2, and AT hook motifs. The SETMAR DBD dimerizes through interactions of HTH1. Residues that interact with DNA are shown as space filling models. A semitransparent surface model (*pink* for the C chain and *cyan* for the B chain) is shown along with a stick model for the TIR-DNA. *B*, the 25-mer DNA duplex derived from the Hsmar1 TIR used for cocrystallization with SETMAR DBD is shown with its core 19 bp binding element shaded in *gray*. A schematic diagram of SETMAR DBD is shown above with the relative positions of HTH1 and HTH2 motifs. *Red letters* are sequence-specific interactions identified from the crystal structure. Nucleotides are numbered according to the TIR complex monomer structure. *C*, close-up view of base-specific contacts made by R371 in HTH1 motif. Two hydrogen bonds are formed between the side chain and the base bG5 (position 5 guanine of chain b, see Fig. 1*B* for number scheme). The DNA is shown as a stick model with a semitransparent surface model in *light gray* in (*C*–*E*). *D* and *E*, close-up view of base-specific contacts in HTH2 motif of TIR complex. Key residues (R417, H427, S428, and R432) make seven hydrogen bonds with DNA nucleobases. Details of AT hook interactions involving R392 and R395 are shown in Fig. S3. DBD, DNA-binding domain; HTH, helix–turn–helix; TIR, terminal inverted repeat.

The structure of the complex is dimeric with two DBDs, each bound to a TIR-DNA duplex oriented in parallel to one another; the DBD comprises two helix–turn–helix (HTH) motifs connected by a 17 amino acid residue linker (residues 384–400) containing two AT hook elements bound to the 25-mer TIR DNA duplex (Figs. 1*A* and S3). The DBD is dimeric as isolated and characterized by gel filtration chromatography (Fig. S1 in (19)). A relatively large surface area (1610 Å^2^) involving interactions of F344, F363, and I341 in each HTH1 motif is buried in the dimer interface (Fig. S2). Sequence-specific recognition of the 19 bp TIR element (5′-GGTGCAAAAGTAATTGCGG) is mediated by 32 hydrogen-bonding interactions, nine in the major groove (two in HTH1 and seven in HTH2), five in the minor groove from the AT-hook elements within the linker, and 18 with the phosphodiester backbone (Figs. 1*B* and S3). Within HTH1, the larger of the two HTH motifs, a single residue, R371 in recognition helix α3, forms two nucleobase-specific hydrogen bonds with G5 (B chain). From HTH2, S428 and R432 of recognition helix α6 hydrogen bond to C18 and G17 (B chain) and G8 and C9 (C chain); R417 and H427 hydrogen bond to G5 and G6 (C chain), respectively (Figs. 1*C* and S3*A*). Within the central AT-rich regions of the TIR, R392 and R395 act as two AT hooks forming specific hydrogen bonds to nucleobases in the minor groove (Fig. S3, *B* and *C*).

**Figure 2:**
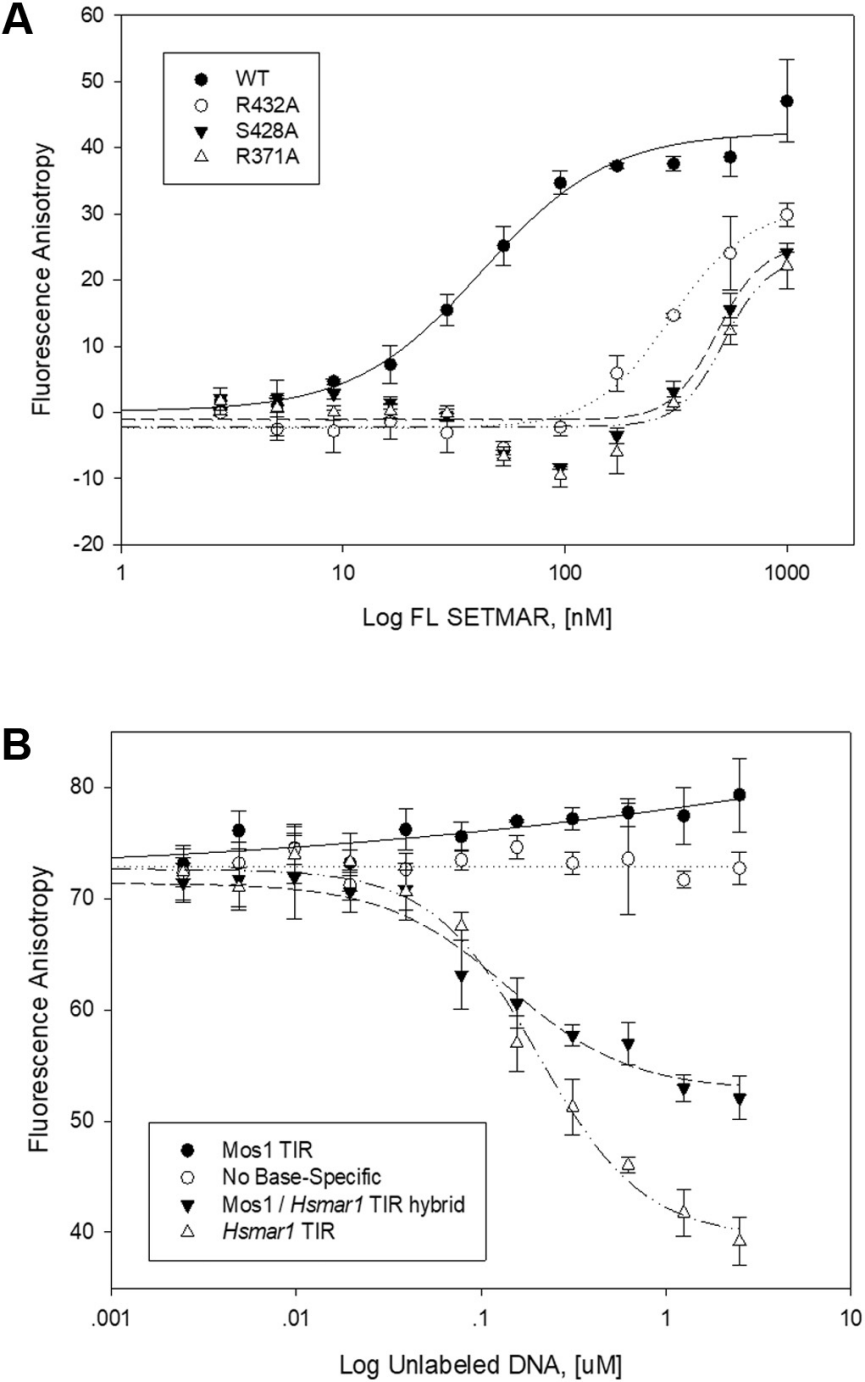
**Key residues and nucleobases direct SETMAR binding to TIRs.***A*, substitutions in key amino acid residues decrease DNA-binding affinity of SETMAR. Rhodamine-labeled TIR probe (10 nM) was titrated with increasing amounts of full-length (FL) SETMAR proteins. Binding curves were fitted for log of protein concentration *versus* fluorescence anisotropy signal. The binding affinity of the mutants is decreased approximately 10-fold compared with that of WT SETMAR. *B*, competition assays using various nonfluorescently labeled DNA sequences. Increasing concentrations of unlabeled Hsmar1 TIR DNA (*empty triangle*) were used to compete off the bound TIR DNA probe from a complex, showing a curve with decreasing fluorescence anisotropy. Mos1 TIR (*filled circle*), a noncognate DNA sequence for Hsmar1 transposase, was unable to compete off the bound TIR DNA probe, serving as a negative control. As a consequence of substitutions of all key nucleotides involved in major groove interactions from G:C to A:T base pairs, a mutant Hsmar1 TIR DNA (*empty circle*) had no measurable competition capability, behaving like the negative control. The error bars indicate the standard deviation of three independent assays with triplicate measurements made in each assay. TIR, terminal inverted repeat.

To assess the contributions of specific residues in the DBD to the overall binding affinity to TIR-DNA, we performed fluorescence anisotropy (FA) assays. Full-length (FL) SETMAR binds with high affinity to the TIR element with a *K*_*D*_ value of 53 ± 4 nM (Fig. S4). Individual substitution of Ala for R371, S428, or R432 located in the recognition helices in FL SETMAR resulted in significant decreases in affinity with relative *K*_*D*_ values for binding of WT, R371A, S428A, and R432A to TIR DNA of 42 ± 5, 521 ± 65, 485 ± 58, and 302 ± 47 nM, respectively (Fig. 2*A*). The finding that R432 is critical for TIR-DNA binding is consistent with previous studies (11, 15). Similarly, an oligonucleotide in which the critical nucleobases were substituted failed to compete effectively for SETMAR binding to the labeled TIR-oligonucleotide in fluorescence polarization competition assays (Fig. 2*B*). Thus, our DNA-binding studies validate the interactions observed in the crystal structure.

In comparing the structures of the SETMAR DBD–DNA complex to related DBD–DNA complexes from MOS1 and TC3, we note that the overall folds of the DBDs of the proteins and dimeric arrangements are similar (Fig. S5). The rmsd for superpositioning of 76 Cα atoms (with rmsds less than 2 Å) in SETMAR and MOS1 using Matchmaker (Chimera (20)) is 1.0 Å and for 106 Cα atoms is 1.9 Å. Matchmaker has the option to prune Cα atoms with rmsds over a cutoff in this case of 2.0 Å to get the best possible match. TC3 is less similar with an rmsd of 1.2 Å for superpositioning with SETMAR of 39 Cα atoms (with rmsds less than 2 Å) and for 4.9 Å for 96 Cα atoms. This is largely because of the fact that HTH1 in TC3 is about 10 residues shorter than the equivalent motifs in SETMAR and MOS1; HTH2 motifs are of similar size in all three proteins. In each complex, a recognition helix from each HTH motif is positioned in the major groove of the DNA; however, the number of amino acids and the type of amino acid involved in nucleobase-specific contacts differ (Figs. S5 and S6). Each complex involves a direct interaction between structurally equivalent but nonidentical HTH1 residues R371, K44, and H37 from SETMAR, MOS1, and TC3, respectively (Fig. S6). Similarly, in HTH2, two structurally equivalent but nonidentical residues are involved in nucleobase-specific contacts; S427/H428, Q100/Q101, and K93/R94 in SETMAR, MOS1, and TC3, respectively. The number of residues involved in nucleobase-specific recognition also differs. For example, in the SETMAR DBD–DNA complex, R371 is the only residue in HTH1 that directly hydrogen bonds to a nucleobase, whereas in MOS1, there are two residues (R44 and R48) and in TC3, three residues (H26, R36, and H37). In HTH2, four residues in SETMAR (R417, H427, S428, and R432), two residues in MOS1 (Q100 and Q101), and two residues in TC3 (K93 and R94) are involved in direct nucleobase interactions. Most of the nucleobase-specific interactions involve guanine (G) nucleobases; however, these Gs are not in structurally equivalent positions in all the structures, and the DNA recognition sequences are different (Figs. S5 and S6). These structural differences suggest that it would not be possible to reliably predict the critical interactions involved in nucleobase-specific recognition in one structure based on the structure of one of the other related protein–DNA complexes.

### SETMAR binds to genomic TIR sequences primarily outside promoter regions

**Figure 3:**
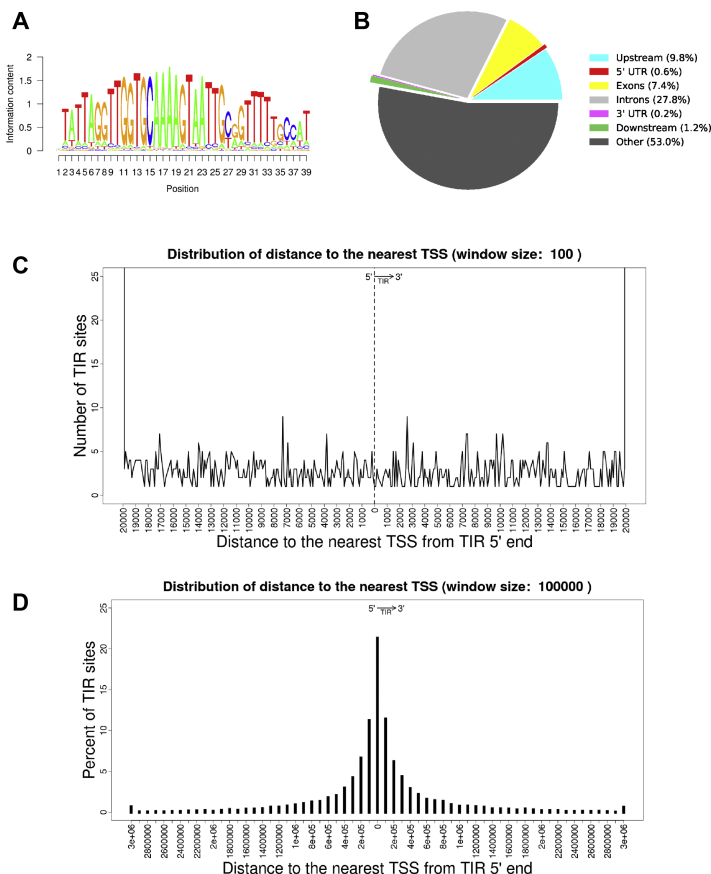
**The preferred genomic-binding site for SETMAR is the ancestral TIR sequence.***A*, the program rGADEM (21) was used to perform a *de novo* motif analysis of SETMAR-binding sites identified through ChIP-Seq analysis; the ancestral TIR sequence was identified as the most common motif bound by SETMAR. The SETMAR-binding sites identified by subtracting the R371A from the WT ChIP-Seq peaks were analyzed using rGADEM to determine a consensus DNA motif for SETMAR binding. Nucleotides 11 to 29 of the consensus motif represent the core 19 bp binding element. The only nucleotide involved in major groove hydrogen bonding interactions for which there is degeneracy is the C/T at position 27. *B*, the program PAVIS (59) was used to analyze the ChIP-Seq data; the majority of SETMAR-binding sites are located in intergenic regions (56.6%) or introns (33.7%). Only 9.8% of sites are located within 10,000 base pairs or less upstream of a transcription start site. *C*, the distribution of distances to the nearest TSS from the 5′ end of the TIR sites with two or fewer mutations. Distances were capped to 20,000 bp shown in 100 bp bins. *D*, distribution of the distances to the nearest TSSs from the 5′ end of TIR sequences. The distribution of distances to the nearest TSS from the 5′ end of the best TIRs (those identified as matches using a PWM) is plotted in 100 kb bins and exhibits a central peak with ∼22% of TIRs falling within 100 kb of the closest TSS. ChIP-Seq, chromatin immunoprecipitation sequencing; PWM, position-weighted matrix; TIR, terminal inverted repeat; TSS, transcription start site.

We took advantage of insights gained from the structural and biochemical assays described previously to generate reagents that would allow us to identify specific SETMAR interactions within the genome. High-quality ChIP grade affinity reagents for SETMAR were not available, so we opted to use WT and DNA-binding mutant (R371A) versions of FLAG-tagged SETMAR, which we transiently overexpressed in human embryonic kidney 293T (HEK293T) cells. The ChIP experiment was validated using a perfect TIR sequence upstream of the CDC23 gene (Fig. S7). Using this approach, we identified sequences bound by FLAG-tagged SETMAR within the human genome of HEK293T cells by next-generation sequencing (ChIP-Seq). Peaks identified from cells expressing similar levels of a DNA-binding mutant FLAG-tagged R371A SETMAR, which has significantly reduced DNA-binding activity, were subtracted from those obtained for WT FLAG-tagged SETMAR removing nonspecific binding from the analysis. Using these criteria, a total of 7323 ChIP peaks were identified for SETMAR (Table S2). *De novo* sequence motif analysis was performed using the program rGADEM (21); the only significant sequence identified was an exact *Hsmar1* TIR sequence (Fig. 3*A*). Within our ChIP data, ∼70% of the peaks include identifiable TIR sequences: 720 with perfect matches, 2361 with one mismatch, and 1352 with two mismatches. We conclude that the preferred binding site of SETMAR in the human genome is the ancestral *Hsmar1* TIR sequence. This finding is in agreement with a recent report that also identified the *Hsmar1* TIR as the preferred binding site for SETMAR in HAP1 cells (22) and contrasts with previous reports (6, 7) in which alternative sequence motifs were identified.

To assess possible roles for SETMAR–TIR interactions, we analyzed the genomic locations of the TIR sequences recognized by SETMAR. SETMAR-bound TIRs are found on all chromosomes, primarily in intergenic (53%) and intronic (27.8%) regions; 9.8% are located within 10 kb of a transcription start site (TSS) (Fig. 3*B*). Binding of typical transcription factors results in a relatively narrow peak close to −200 bp from the TSS (23). In contrast, despite the fact that ∼10% of TIRs are within 10 kb of TSSs, the distribution of SETMAR-bound TIRs within 20 kb of TSSs is broad and lacks a defined peak (Fig. 3*C*). Instead, ∼22% of the best SETMAR-bound TIR sequences (identified through the use of a position weighted matrix analysis) are found within 100 kb of TSSs (Fig. 3*D*).

**Figure 4:**
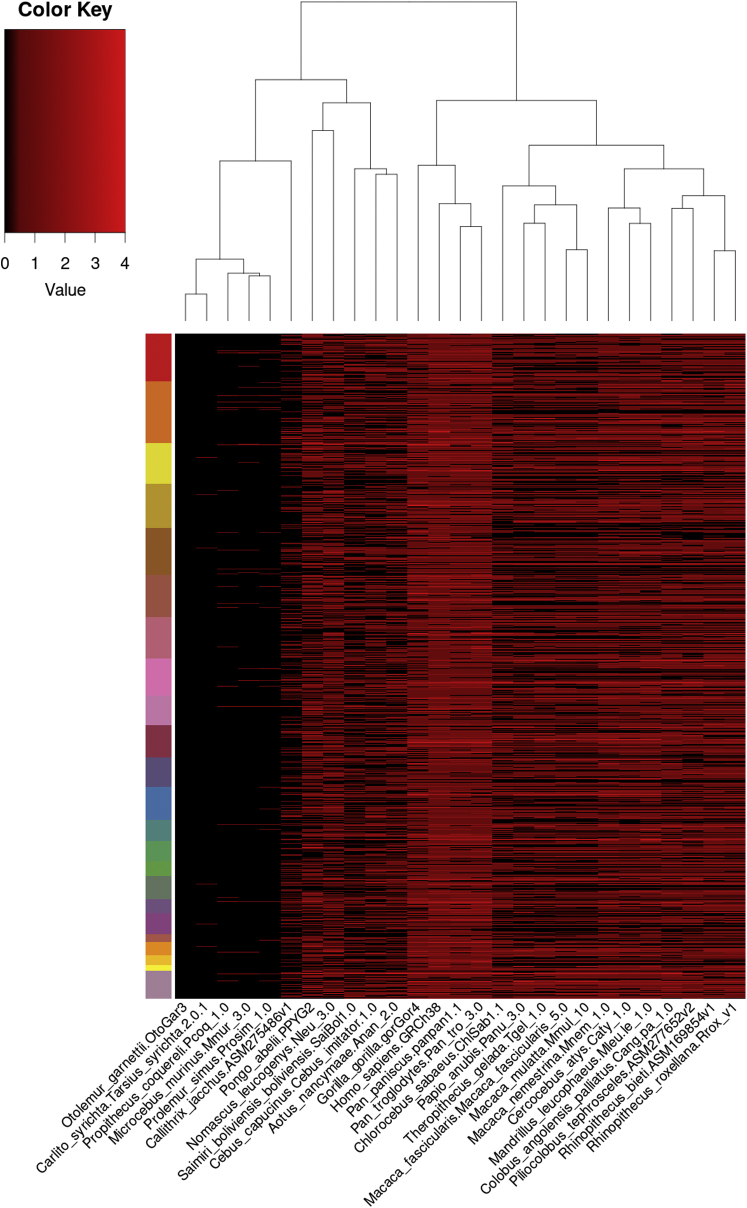
**A position-weighted matrix was generated from all SETMAR-bound TIR sequences with two or fewer mismatches compared with the perfect 19-base pair sequence identified by ChIP-Seq in human HEK293T cells.** Using the PWM, 5253 TIR sequences were identified in the human genome, 763 of which were perfect, 2361 had one mismatch, and 1352 had two mismatches. The heat map shows the distribution of SETMAR motif ChIP peak hits among the human Genome Evolutionary Rate Profiling (GERP) constrained elements across the 27 selected primates. Each row in the heat map represents a block of human genome sequence containing all those GERP constrained elements that are not farther than 10 Kb from the adjacent ones (http://ftp.ensembl.org/pub/release-98/bed/ensembl-compara/90_mammals.gerp_constrained_element/). These blocks were sorted on their positions on the chromosomes ordered 1 to 22, X and Y, each represented by a different color on the *left side bar*. The data represent the number of SETMAR motif ChIP peak hits within each such block in the human genome and the corresponding homologous regions in the other selected primate genomes as indicated on the color key. This clustering recapitulates current phylogenetic classification of primates. ChIP-Seq, chromatin immunoprecipitation sequencing; HEK293T, human embryonic kidney 293T cell line; PWM, position-weighted matrix; TIR, terminal inverted repeat.

If SETMAR functions through interactions with genomic TIR sequences, then we would expect these TIR sequences to be conserved in other simian primates, which are the only species other than humans that have SETMAR. To analyze genomic TIR sequences, we created a position-weighted matrix (PWM) derived from the TIR sequences contained within our ChIP peaks with two or fewer mismatches to the 19 bp SETMAR recognition element 5′-GGTGCAAAAGTAATTGCGG (Table S3). These TIR sequences and their respective genomic locations served as the reference for this analysis. For the 27 primate genomes available, TIR sequences and their respective genomic locations were then clustered by degree of conservation with the human genome (Fig. 4). Strikingly, the TIR sequences within simians, catarrhine and platyrrhine primates, are conserved, with the most closely related genera (*homo*, *gorilla*, and *pan*) having a more similar number and distribution of TIR sequences followed by the next most closely related (*pongo* and *nomascus*) and then less closely related primate genera. These results are consistent with the current evolutionary clustering of primates (24) and strongly suggest that both SETMAR and TIR sequences have been conserved for function in simiiformes.

### Loss of SETMAR alters the transcriptome

HEK293T cells express two variants of SETMAR as visualized on a Western blot probed with a SETMAR-specific antibody: FL SETMAR (∼80 kDa) and a previously reported splice variant (VarA) (25, 26) (∼50 kDa) (Fig. S8*A*). VarA results from expression of a splice variant including exon 1, which encodes the N-terminal 52 amino acid residues of the SET domain, and exon 3, the mariner transposase, including DBD and catalytic domains. Our SETMAR expression results are consistent with those reported in gliobastoma-derived cells (26) and in acute myeloid leukemia patient samples (25), in which both FL and a stable splice variant referred to as SETMAR-1200 or VarA, respectively, were identified. Our results contrast with a recent study focused on colon cells in which a number of truncated variants but no FL SETMAR were identified (7). The SET catalytic domain is encoded by exon 2; thus, VarA lacks lysine methylation activity associated with the SET domain and retains only the MAR functions. To create a KO cell line, we used a double nickase CRISPR/Cas9 strategy (27, 28) with two guide RNAs to target Cas9 to the first exon of SETMAR in HEK293T cells. Western blot and sequencing analyses confirmed that both FL SETMAR and VarA were absent in KO clones (Fig. S8*B*).

**Figure 5:**
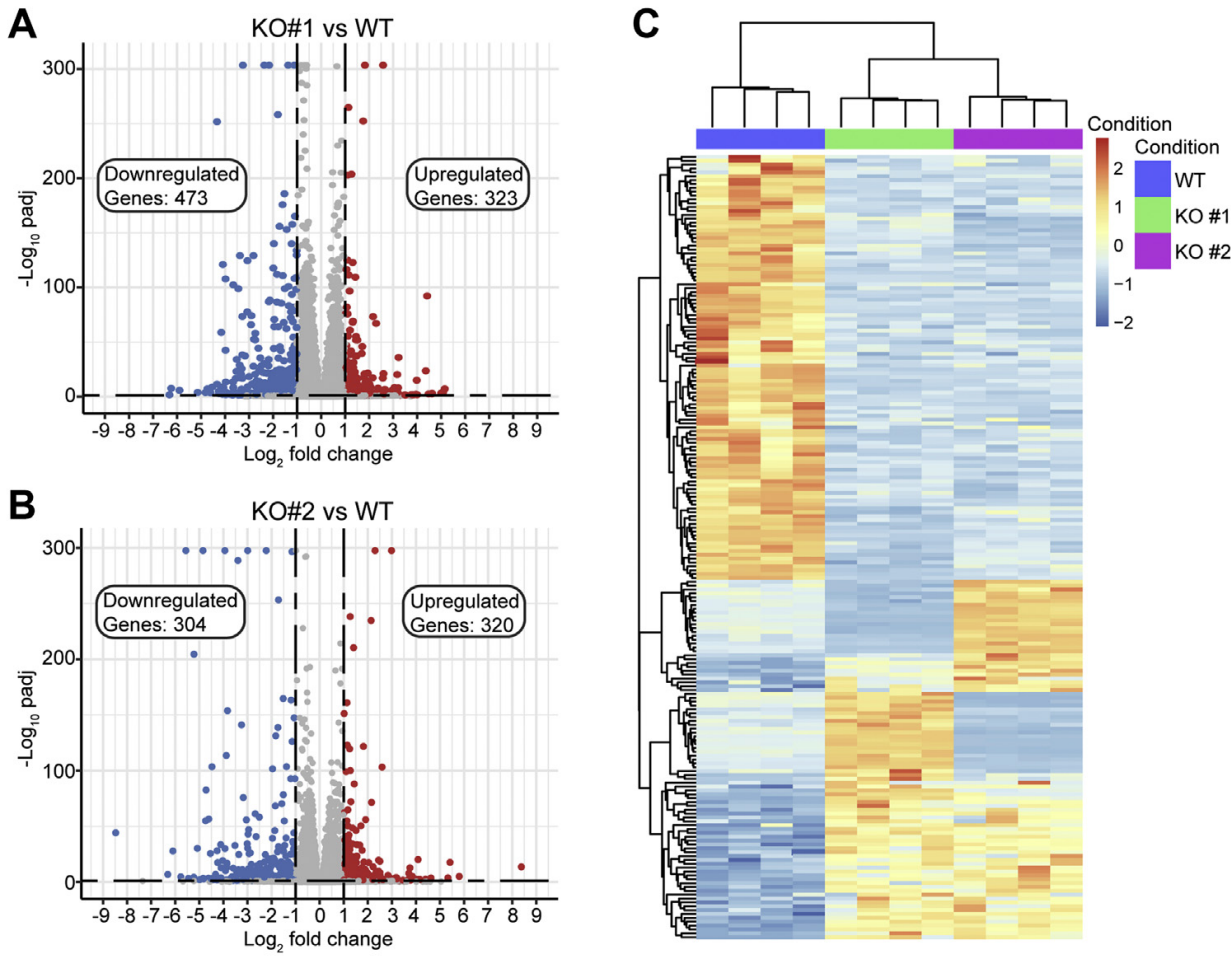
**Loss of SETMAR expression changes the transcriptome.***A* and *B*, volcano depicting all transcripts detected compared with WT HEK293T cells. Significantly (Log_2_ fold change >1; padj <0.05) upregulated and downregulated transcripts are colored in *red* and *blue*, respectively. *C*, heat map depicting normalized counts transformed into z-score by row for the 203 differentially expressed genes in common between both KO clones. About 80% of the differentially expressed genes are either upregulated or downregulated in both KO clones relative to the parental cell line. HEK293T, human embryonic kidney 293T cell line.

To determine what impact loss of SETMAR might have on the transcriptome, we performed RNA-Seq analysis. We identified 203 common differentially expressed (DE) genes (false discovery rate [FDR] <0.05 and absolute fold change of 2) in the two SETMAR KO clonal cell lines as compared with the WT parental cells (Fig. 5 and Table S4). Of the DE transcripts identified for SETMAR KO cells, 53 were upregulated and 110 downregulated *versus* parental cells (Table S4), thus a total of 163 genes with changes in the same direction. Among the downregulated transcripts are two genes involved in pre-mRNA splicing (*RBM24* and *CELF4*), six transcription factors (*PAX1*, *SOX21*, *ZNF544*, *SOX3*, *TLX2*, and *ZNF334*), and 11 genes associated with neuronal function (*LMO3*, *NR0B1*, *FLRT2*, *LRFN5*, *NRSN1*, *NEGR1*, *TTPA*, *BAI1*, *NPFFR2*, *GAP43*, and *STXBP5*L). Gene Ontology enrichment (enrichGO) analysis of common 163 DE transcripts indicates a role for SETMAR in a number of biological processes and cellular compartments involving synapses (Fig. S9), specifically synapse organization or assembly and regulation, and synaptic and postsynaptic membranes or density membranes. Overlap of DE genes identified in this study with those reported for overexpression of SETMAR in U2OS cells is limited to a total of nine upregulated and seven downregulated genes of 953 upregulated and 497 downregulated genes identified in that study (6).

### Loss of SETMAR impacts AS

We next addressed the hypothesis that SETMAR facilitates alternative pre-mRNA splicing. Several considerations support this hypothesis. AS is known to be critically important for development in higher organisms (17). And, since SETMAR is a simian-specific protein, a role for SETMAR in AS might reveal a previously unknown influence on primate evolution. A known splicing factor, snRNP70, which is part of the U1 spliceosomal complex, has been reported to be methylated by SETMAR (16). Although the impact of this post-translational modification has not been studied, the most common molecular function of lysine methylation is modulation of protein–protein interactions (29), which in this case could be important for the U1 spliceosomal complex. CTCF, a sequence-specific DNA-binding factor involved in maintaining the 3D structure of chromatin, was shown to impact AS by stalling RNA polymerase II elongation (30, 31). We hypothesize that SETMAR may similarly regulate AS through recognition of TIR sequences.

**Figure 6:**
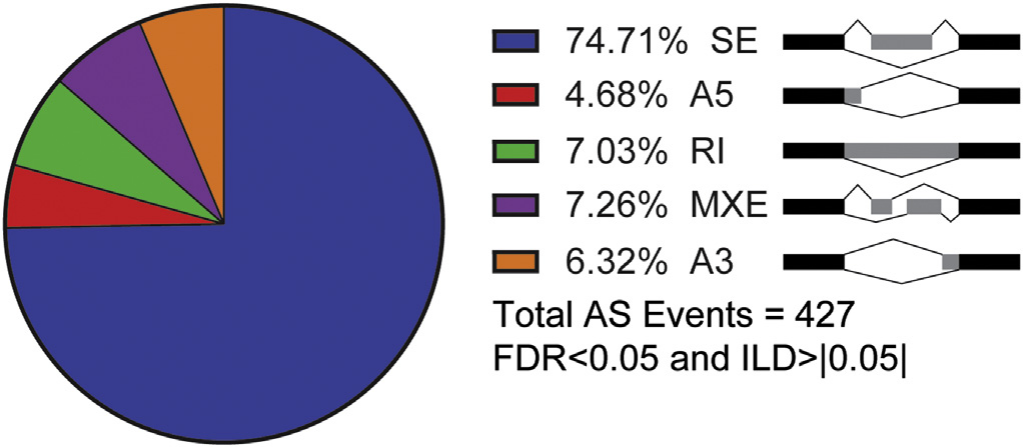
**Loss of SETMAR expression alters alternative splicing (AS).** The pie chart and schematic diagram depict the distribution of 255 AS events common between both SETMAR KO clones.

AS events were examined in the two SETMAR KO cell lines and compared with the parental cells. Differential splicing events were identified as those with an FDR <0.05 and inclusion level differences (ILDs) of less than or greater than 0.05 (*i.e.*, differences of 5% or greater in the number of reads for a specific AS event) (Fig. 6). In this analysis, 255 shared AS events were identified, of which 233 exhibited ILDs with the same sign (either positive or negative) for each KO cell line as compared with the parental cells. The largest category of AS events is skipped exons, with 158. This number of AS events is consistent with a role for SETMAR in regulating AS. There is no overlap in the lists of genes found to be alternatively spliced and those differentially expressed in the comparisons of parental and KO cell lines.

SETMAR alters AS events for a number of different genes including those associated with transcription, splicing, and neuronal function (Table S5). Several genes were modified by at least two types of AS events (*ARHGEF40*, *CAST*, *CCDC24*, *CHKB*, *FAM498*, *GRB10*, *MTA1*, *PLOD2*, *PTGR2*, *SLC2A11*, and *ULK3*). Of the AS genes, 16 encode transcription factors/cotranscriptional factors, including *FOXM1*, a regulator of expression for cell cycle genes critical for DNA replication and mitosis, 10 zinc finger proteins, two of which have been characterized, *ZFPM2* and *ZNF692*, and three PRDM (PRDI-BF1 and RIZ homology domain containing) proteins that are likely involved in transcription regulation. Two genes involved in pre-mRNA splicing include *LSM4*, involved in the U4/U6–U5 tri-snRNP complex (32, 33), and *U2AF1* (34). AS genes that were reported to be associated with neuronal function include *ABHD14A*, *GABRD*, *GRIPAP1*, *HUWE1*, *PTPRD*, *TMEM25*, and *TMEM14B*. This latter gene, *TMEM14B*, encodes a primate-specific protein involved in cortical expansion and folding in the developing neocortex. *TMEM14B* marks basally located radial glia, which contributes to evolutionary expansion, and drives growth of neural progenitor populations, and *TMEM14B* is a primate-specific gene that has been postulated to drive neurodevelopment important for evolution of the brain (35). Thus, changes to the transcriptome mediated by SETMAR including differential expression of two known pre-mRNA splicing factors, *RBM24* and *CELF4*, and AS of a number of proteins associated with neuronal function may play an important role in primate development.

### Possible mechanisms for impact on the transcriptome

To examine the underlying mechanism by which SETMAR regulates gene expression and AS, we first considered whether the SET domain function of SETMAR dimethylates H3K36. A previous report found that the KMT activity of SETMAR was required for its impact on gene expression. However, the prior examination of changes in H3K36me2 at specific DE genes was inconclusive (6). Consistent with the proteomic study by Carlson and Gozani (36), we find no evidence of histone methylation within nucleosome substrates; HeLa, chicken, and recombinant nucleosomes were used in our study (Fig. S8*E*, KMT assay). We did find evidence for automethylation activity and methylation of free H3 histones, as previously reported (36). In this case, we assume that the methylation of H3 occurs at K115 as previously suggested (36). Furthermore, we did not observe a global decrease of H3K36me2 in KO cell lines as would be expected if H3K36 is indeed a substrate for SETMAR. In fact, there was a trend toward a slight increase in H3K36me2 in KO cells as compared with the parental cells (Fig. S8, *B* and *C*). This modest increase may result from downregulation of *RIOX1*, an H3K36 demethylase (37, 38), in SETMAR KO cells (Table S4). Finally, it was previously shown that overexpression of NSD2, a *bona fide* H3K36 methyltransferase, results in a global increase in H3K36me2 (39). We overexpressed SETMAR at different levels and found no change in H3K36me2 levels (Fig. S8*D*). Overall, we confirm that it is unlikely that H3K36 is methylated directly by SETMAR in cells. However, SETMAR was reported to methylate snRNP70 and may methylate other proteins that impact DE or AS genes.

A second possibility is that interactions between SETMAR and TIRs may directly impact DE or AS genes. Binding of SETMAR to a TIR within a promoter element might be expected to impact expression of that gene. To assess this possibility, we identified ChIP peaks located within 10 kb upstream of TSSs of DE genes (Fig. 3*C*). Among DE genes, a single TIR is located upstream of a downregulated Y RNA gene. Y RNAs are small noncoding RNAs that have been shown to be essential for initiation of chromosomal DNA replication (40).

A third possibility is that SETMAR impacts expression through binding to enhancer elements. To assess this possibility, we examined the overlap of TIRs that we expect SETMAR to bind with high affinity and known enhancer sites. Using enhancer elements taken from FANTOM5 (32,693) and the Enhancer Atlas (21,417), we identified 236 and 146 enhancer elements, respectively, from each database, that overlap with the best TIRs (Fig. S10). The overlap of TIRs with enhancers is greater than chance by a factor of 1.5 to 2.0. It is possible that SETMAR interactions with TIRs located close to enhancer elements may impact the formation of promoter–enhancer loops that regulate expression of DE genes.

AS of genes may result from binding of SETMAR to TIRs within introns. To assess this possibility, we identified TIRs within introns of AS genes. TIRs identified by ChIP-Seq are present in 10% of the 233 common AS genes. We suggest that direct binding to TIR sequences may explain a portion of AS and DE genes observed in the absence of SETMAR.

## Conclusions

This study addresses the mechanisms by which SETMAR functions as a sequence-specific DNA-binding protein through a combination of crystallographic, biochemical, and genomic analyses. We define how SETMAR recognizes TIR sequences, its affinity for TIRs, which TIRs are recognized by SETMAR in the genome, and which TIRs are conserved in other simian primates. We suggest that SETMAR may alter differential expression and AS through a combination of binding to TIRs and/or methylating lysines in nonhistone protein substrates including snRNP70. Differential regulation of RBM24 and CELF4 transcripts by SETMAR may directly impact AS, and this impact may be further augmented by differences in AS of other splicing factors including LSM4 and U2AF.

Thus, our findings represent an expansion of our current understanding of the impact of TEs in shaping eukaryotic genomes, in this case specifically simian primate genomes, and broadly define a potential role for SETMAR–TIR interactions throughout the genome in the regulation of gene expression and AS of a number of important genes including splicing factors, transcription factors, and neuronal factors, albeit only in simian primates. Specifically, AS of *TMEM14B*, which has been proposed to be the single primate factor identified in a search for proteins involved in cortical expansion and folding, may be an important driver for evolutionary conservation of TIR-specific DNA-binding activity associated with SETMAR.

## Experimental procedures

Throughout this article, we have retained the original numbering scheme for a 671 amino acid residue SETMAR. It has since been reported that the N terminus includes an additional 13 residues (National Center for Biotechnology Information entry: NP_006506.3).

### Protein expression and purification

A variant of the SETMAR DBD including residues 329 to 440 (C381R) along with other variant sequences were expressed in *Escherichia coli* and purified as previously described (19, 41). The DBD protein for crystallization was expressed as an N-terminal His-tagged SUMO fusion protein. The His-SUMO tag was removed *via* on-column cleavage while bound to nickel–nitrilotriacetic acid resin with the SUMO-specific Ulp1 protease and further purified by ion exchange and size-exclusion chromatography as previously described (19).

For biochemical studies, the FL SETMAR (WT) gene was cloned into the NdeI/XhoI site of pET15b (EMD Millipore). Into this plasmid, mutations resulting in R371A, S428A, and R432A variants of the encoded FL SETMAR protein were generated individually by using the QuikChange II site-directed mutagenesis kit (Agilent Technologies). Primers used for PCR amplification (Integrated DNA Technologies, Inc) are as follows. R371A 5′-GCCCAGGAACTGCTAACGAAGCTACAGTGCAGTGG-3′, S428A 5′-GAACTCAATGTCAACCATGCTACGGTCGTTCGACATT-3′, and R432A 5′-ACCATTCTACGGTCGTTGCACATTTGAAGCAAATTGG-3’. Plasmids were verified by DNA sequencing (GENEWIZ, Inc).

FL SETMAR (WT) and mutants were expressed in *Rosetta* cells (EMD Millipore) and induced by culturing at 20 °C overnight with 0.1 mM IPTG and 50 μM ZnCl_2_ (16). Cells were lysed in a solution of 50 mM phosphate, pH 7.8, 300 mM NaCl, and 10 mM imidazole by French press (Aminco), and the sample was clarified by ultracentrifugation at 35,000 rpm for 30 min at 4 °C. Purification included nickel–nitrilotriacetic acid, Q-Sepharose, and size exclusion (Superdex 200 16/60) chromatographic separations. FL SETMAR (WT) and substituted proteins were concentrated using 10 kDa molecular weight cutoff concentrators (EMD Millipore). The proteins were stored in a solution of 50 mM Tris-Cl (pH 7.0), 500 mM NaCl, and 1 mM DTT at −80 °C.

### DNA oligonucleotides for crystallization

For the TIR complex, two oligonucleotides, 5′-GGTTGGTGCAAAAGTAATTGCGGTTA-3′ and its complementary strand 5′-AACCGCAATTACTTTTGCACCAACCT-3′, were annealed to form a 25-mer duplex DNA with overhanging 3′ A and T, respectively. For experimental phasing, the underlined “Ts” were replaced by 5-bromodeoxyuridine (5-BrdU). All oligonucleotides were gel-purified 26-mers purchased from Midland Certified Reagent Company, Inc.

### Crystallization

As previously reported, the DBD was crystallized with a perfect TIR sequence to form the protein–DNA complex (19). In brief, DBD protein was mixed with duplex DNA (5 mM stock) to make a final protein:DNA molar ratio of 1:1.2 in a solution of 50 mM Hepes (pH 7.5), 150 mM NaCl, and 1 mM DTT. The resulting protein concentration was 500 μM. The protein–DNA complex was incubated on ice for 15 min prior to crystallization. Initial crystals were grown by vapor diffusion in hanging drops at 20 °C. The reservoir solution contained 0.1 M magnesium formate and 15% PEG3350. Crystals for data collection were obtained by microseeding, cryocooled in a solution containing 20% ethylene glycol, 0.1 M magnesium formate, and 8 to 15% PEG3350, and flash frozen in liquid nitrogen before data collection.

### Data collection and data processing

Diffraction data were collected at 100 K at the 23-ID-B, 23-ID-D, and 19-ID beamlines at the Advanced Photon Source, Argonne National Laboratory. For experimental phasing, single-wavelength anomalous diffraction (SAD) datasets were collected from BrdU-labeled or BrdU/SeMet-substituted protein–DNA complex crystals at the bromine or selenium (Se) absorption peak wavelength, 0.91922 and 0.97938 Å, respectively. Optimal crystals were grown using the BrdU oligonucleotides. Diffraction data were processed using XDS (42) at 23-ID beamlines or HKL3000 (43) at 19-ID. Statistics for data processing and crystallographic refinement statistics are summarized in Table S1.

### Experimental phasing and structure determination

Se-SAD data (TIR complex; Table 1) were collected to 2.66 Å for DBD 329 to 440 (C381R) (I359M) (L423M) complexed to BrdU-substituted TIR DNA. Details of the experimental phasing strategies have been reported (19). In brief, using AutoSol (44), a total of five Se sites were identified; phases calculated from these sites resulted in a very interpretable electron density map. Autobuild functions within AutoSol (44) were used to obtain a partial model of the DNA and two HTH motifs. A model containing amino acid residues 334 to 437 and the entire DNA duplex was completed through model building in *COOT* (45). The positions of the SeMet residues were confirmed by anomalous difference Fourier analysis (Fig. S2).

**Table 1:** DNA oligonucleotides for competition assays

Name	Sequence (5′-3′)
*Hsmar1* TIR	TTAGGTTGGTGCAAAAGTAATTGCGGTT
Mos1 TIR	TCAGGTGTACAAGTATGAAATGTCGTTT
*Hsmar1*/Mos1 TIR hybrid	TTAGGTTGGTGC**GTATGA**AATTGCGGTT
*Hsmar1* TIR without sequence-specific–binding sites	TTAGGTT**AA**T**AT**AAAAGTAATTG**T**GGTT

The underlined bases are mutated from the *Hsmar1* TIR sequence.

Diffraction data for the TIR complex (TIR complex, high resolution in Table S1), in which residues 329 to 440 with C381R, I359M, L423M substitutions complexed with brominated TIR DNA, were collected to 2.37 Å. The structure was determined by molecular replacement in PHASER (46) using the initial structure derived from the experimental Se-SAD phasing as the search model. A final refined model was obtained following iterative cycles of model building in *COOT* (45) and refinement in PHENIX (44) and BUSTER (47) using individual atomic coordinates and *B*-factors, maximum likelihood targets, and TLS parameters. Based on analysis from the TLS Motion Determination server (http://skuld.bmsc.washington.edu/∼tlsmd/index.html), the TIR complex was partitioned into six TLS groups: chain A (331 to 396), chain A (397 to 437), chain B (1 to 15), chain B (16 to 26), chain C (1 to 10), and chain C (11 to 26).

### FA assay

FA assays were conducted as previously described (41). A 5’-(rhodamine) (C6 amino)-AACCGCAATTACTTTTGCACCAACCTAA-3′ oligonucleotide was annealed to its complementary sequence to make the *Hsmar1* TIR duplex DNA probe. In brief, 20 nM rhodamine-labeled DNA probe was incubated with varying concentrations of protein in a 50 μl reaction mixture buffered in 50 mM Hepes (pH 7.0), 150 mM NaCl, and 1 mM DTT. Oligonucleotides were ordered from Midland Certified Reagent Company, Inc. FA data were measured by using the Envision 2102 Multilabel Plate Reader (PerkinElmer Life Science) in the Chemical Genomics Core Facility of Indiana University School of Medicine. *K*_*D*_ values were calculated by fitting the data to a one-site binding saturation ligand-binding curve (SigmaPlot, version 11.2). Three independent experiments were conducted for each titration, each with triplicate measurements.

### Protein–DNA binding competition assay

Competition assays were performed by titrating preformed protein–rhodamine DNA solution with an unlabeled DNA duplex. Duplexes that successfully competed for binding to SETMAR displaced the fluorescent probe resulting in a loss of FA. About 300 nM FL SETMAR (WT) with 20 nM DNA probe was used in these assays. The concentrations of protein and DNA probe were determined from the binding assay in which 70% of the saturated FA was measured. The buffer used in this experiment is the same as aforementioned, 50 mM Hepes (pH 7.0), 150 mM NaCl, and 1 mM DTT. The data were plotted as a function of anisotropy against log-unlabeled DNA concentration. The DNA sequences used in the study are shown in Table 1.

### Cell culture

HEK293T cells were cultured in Dulbecco’s modified Eagle’s medium supplemented with 10% fetal bovine serum (Atlanta Biologicals) and 1% penicillin/streptomycin (Hyclone; GE Healthcare Life Sciences) in an incubator at 37 °C and 5% CO_2_. Analysis of H3K36me2 with a titration of SETMAR expression was performed by transfecting HEK293T cells grown to ∼70 to 80% confluence in a 6-well dish with 0.25, 0.5, and 1 μg of pFLAG-cytomegalovirus 4 (CMV4)-SETMAR or pFLAG-CMV4 (empty vector) using XtremeGENE 360 (Roche). After 24 h, cells were harvested by trypsinization, washed with PBS, and resuspended in lysis buffer (10 mM Pipes [pH 7.0], 300 mM sucrose, 100 mM NaCl, 3 mM MgCl_2_, 0.1% Triton X-100, 1× Universal Nuclease, and 1× Protease Inhibitor Cocktail from Thermo). Total protein was quantified by Bradford Assay (Bio-Rad). Samples were resolved by SDS-PAGE, transferred to polyvinylidene difluoride membrane (Thermo), and probed with the indicated antibodies (FLAG [Sigma; catalog no.: F1804]; β-tubulin [Proteintech; catalog no.: 66240-1]; SETMAR [Proteintech; catalog no.: 25814-1-AP #05–661]; Histone H3 [Cell Signaling Technologies; catalog no.: 9715]; and H3K36me2 [Cell Signaling Technologies; catalog no.: 2901]).

### ChIP DNA preparation

HEK293T cells obtained from American Type Culture Collection were seeded into four 10 cm dishes, each with 2.5 million cells. At 70 to 80% confluency, cells were transfected with pFLAG-CMV4-SETMAR (WT) or with pFLAG-CMV4-SETMAR (R371A) at the amount of 20 μg per dish using the polyethyleneimine transfection protocol. After 20 h, the transfected cells were fixed in 1% formaldehyde (catalog no.: 28906; Thermo Scientific). Crosslinking of proteins to DNA was allowed to occur at room temperature for 10 min and was stopped by adding glycine to cells at a final concentration of 125 mM. Cells were lysed in a solution containing 50 mM Tris (pH 7.5), 150 mM NaCl, 5 mM EDTA, 1% Triton X-100, 0.5% SDS, and protease inhibitors (1 mM PMSF, 1× protease inhibitor cocktail Set V [catalog no.: 539127; EMD Millipore]). DNA was sonicated to an average length of 150 bp using a Bioruptor 300 (Diagenode) device. For each dish of cells, the sonication setting was 30 s ON and 30 s OFF for each cycle, total 60 cycles at high power.

Since SETMAR was overexpressed with a FLAG tag at the N terminus, immunoprecipitation was performed using anti-FLAG M2 affinity agarose (catalog no.: A2220; Sigma–Aldrich). Before immunoprecipitation, 40 μl (50% slurry) was washed with buffer containing 50 mM Tris (pH 7.5), 150 mM NaCl, 5 mM EDTA, and 1% Triton X-100. For immunoprecipitation, at least 50 μg sheared chromatin DNA was added to the washed agarose beads and rotated overnight at 4 °C. After incubation, agarose beads were washed three times in low salt wash buffer (50 mM Tris [pH 7.5], 150 mM NaCl, 5 mM EDTA, and 1% Triton X-100), one time in high salt wash buffer(50 mM Tris [pH 7.5], 500 mM NaCl, 5 mM EDTA, and 1% Triton X-100), and then one time in Tris EDTA buffer (pH 8.0). The ChIP complexes were eluted by incubating with 150 μl of 0.1 M glycine (pH 3.5) for 5 min at room temperature, followed by neutralization with 15 μl of solution of 0.5 M Tris (pH 7.5), and 1.5 M NaCl. To reverse crosslinks and digest protein, 2 μl of proteinase K (20 mg/ml; catalog no.: AM2546; Ambion) was added, and the mixture was incubated at 65 °C for 2 h. ChIP DNA samples were purified by using a PCR purification kit (Qiagen) and eluted with 30 μl elution buffer in the final step. These ChIP DNA samples were then used for quantitative PCR (qPCR) and ChIP-Seq analysis.

### ChIP–qPCR assay

To validate the ChIP-Seq result, eluted ChIP DNA was quantified using qPCR. To detect the TIR-binding site upstream of *CDC23* gene, ChIP DNA and 2% input DNA were quantified using qPCR. PCR mixtures contained 5 μl of ChIP DNA, 2 μl of primer pairs (10 μM), 10 μl of 2× SYBR-Green Reaction Mix (Bioline USA, Inc), and 3 μl of double-distilled water in a total volume of 20 μl. Primer pairs are 5′-ACCTAAAGGCAAACTCTAACTCCA-3′ and 5′-ACTGTACTCCAGCCTGGTCAA-3′, which are flanking the TIR site upstream (about 6000 bp) of *CDC23* gene. qPCR was performed at 95 °C for 3 min, 40 cycles of denaturation (95 °C for 15 s), and annealed/extended at 60 °C for 60 s. Amplification and detection were measured on the Realplex2 Master Cycler (Eppendorf). The signal ratio of ChIP DNA to input DNA (percent input of ChIP) was calculated by using 2% X 2ˆ(C_T_ 2% input DNA sample – C_T_ ChIP DNA sample). Results were obtained from three independent ChIP experiments with three technical replicates each.

### ChIP-Seq analysis

ChIP DNA samples from SETMAR (WT) and SETMAR (R371A) groups were sequenced by the Center for Medical Genomics at Indiana University School of Medicine using an Illumina HiSeq 4000 system. Paired-end sequences were aligned to the human genome (hg19) using bowtie2-2.2.6. SETMAR peak locations were determined using the MACS software (version 2.1.1.20160309) with a cutoff of *P* = 5e-5. ChIP peaks were remapped to hg38 using the University of California Santa Cruz (UCSC) liftover tool. The motif analysis program rGADEM (21, 48) was used to discover the consensus DNA sequence for SETMAR binding. DNA sequences bound by SETMAR during ChIP were derived by analyzing ChIP-Seq-binding site locations using the GenomicRanges Bioconductor package in R (49).

### Conservation in primate analysis

These ChIP-Seq peak sequences were then searched for the consensus *Hsmar1* TIR sequence motif (GGTGCAAAAGTAATTGCGG) using FIMO (Find Individual Motif Occurrences) web software (50). From 4433 unique ChIP peaks that contained TIR sites defined as the 19 bp motif that interacts directly with SETMAR (5′-GGTGCAAAAGTAATTGCGG) with two or fewer mismatches, a PWM of log-likelihood ratios for each nucleotide at each base position of the motif. The likelihood ratio for each nucleotide at a given base position was calculated by dividing the corresponding nucleotide frequency at that position with the nucleotide's background frequency estimated from the human genome sequence (A = 29.5%, C = 20.4%, G = 20.5%, and T = 29.6%). Sequences for 27 primate genomes were downloaded from ENSEMBL. Loci across these 27 species matching the computed PWM were identified using MOODS (Motif Occurrence Detection Suite), version 1.9.4.1, with the match score threshold set to 28 (-t 28) (51). The SETMAR TIR sites identified with MOODS across all 27 primate genomes were annotated using the corresponding constrained element data available from ENSEMBL (ftp://ftp.ensembl.org/pub/re1ease-98/bed/ensembl-compara/90_mamma1s.gerp_constrained_element/). Sites not farther than 10 kb of any constrained element were associated with that element and the corresponding location on the human genome. The constrained elements on the human genome closer than 10 kb from each other were grouped into blocks, and the number of SETMAR TIR sites from each block from each species was counted. The 27 primate species were clustered hierarchically on these counts.

### CRISPR silencing of SETMAR

SETMAR was silenced in HEK293T cells using the CRISPR/Cas9 double nickase method outlined by Ran *et al.* (27, 28). Two single-guide RNAs (sgRNAs) targeting exon 1 of SETMAR were designed using the tool at https://crispr.mit.edu. (sgRNA1: 5′-TTAAACTCCGCCATCCCACA-3′; sgRNA2: 5′-GAGCAGCTGGATGTCGCGTG-3′). sgRNA 1 was cloned into pSpCas9n(BB)-2A-GFP (PX 461) (plasmid #48140), and sgRNA 2 was cloned into pSpCas9n(BB)-2A-Puro (PX 462) V2.0 (plasmid #62987). Both plasmids were obtained from Addgene. Plasmids were transiently cotransfected using polyethyleneimine transfection into HEK293T cells. About 24 h after transfection, GFP+ cells were isolated by flow cytometry in the Indiana University School of Medicine Flow Cytometry Core and replated. About 24 h after flow sorting, cells were treated with 6 μg/ml puromycin for 72 h. Remaining cells were then separated into single cell clones and grown into clonal cell lines. SETMAR^-^ cell lines were validated by Western blot (anti-SETMAR antibody; catalog no.: 25814-1-AP; Proteintech) as well as by subcloning the PCR-amplified CRISPR target site (forward primer: 5′-ACAAATGACCTCACCTCGAAAG-3′; reverse primer: 5′-TGAGGACAGGACTGGACAAA-3′) into the pCR-4 TOPO-TA vector (catalog no.: 45-0030; Invitrogen), sequencing resulting gene alterations (GENEWIZ), and predicting translation of edited gene product using the ExPASy translate tool (https://web.expasy.org/translate). Western blot analysis using FLAG (Sigma; catalog no.: F1804) and Cas9 (Cell Signaling Technologies; catalog no.: 14697S) antibodies was done to ensure that KO clones had not integrated FLAG-tagged Cas9 present on the plasmids used in the CRISPR/Cas9 process.

### Next-generation RNA sequencing

Cells were seeded into 6-well plates, 500,000 cells per well. Cells were harvested 48 h after seeding, and RNA was isolated and purified using the QIAGEN RNeasy Plus Mini Kit (Qiagen, Inc). Four replicates were prepared per cell type.

Sequencing was performed by GENEWIZ. Concentration and quality of total RNA samples were first assessed using the Agilent 2100 Bioanalyzer. A RNA integrity number of five or higher was required to pass quality control. About 500 ng of RNA per sample were then used to prepare a single-indexed strand-specific complementary DNA library using the TruSeq Stranded mRNA Library Prep Kit (Illumina). The resulting libraries were assessed for quantity and size distribution using Qubit and the Agilent 2100 Bioanalyzer. About 200 pM pooled libraries were utilized per flowcell for clustering amplification on cBot using HiSeq 3000/4000 PE Cluster Kit and sequenced with 2 × 75 bp paired-end configuration on HiSeq4000 (Illumina) using the HiSeq 3000/4000 PE SBS Kit. A Phred quality score (Q score) was used to measure the quality of sequencing. More than 90% of the sequencing reads reached Q30 (99.9% base call accuracy).

The initial mapping and processing of the RNA-Seq data was done by the Center for Computational Biology and Bioinformatics as described later. Sequencing data were assessed using FastQC (Babraham Bioinformatics) for quality control. All sequenced libraries were then mapped to the human genome (UCSC hg38) using STAR RNA-Seq aligner (52) with the following parameter: “--outSAMmapqUnique 60.” The read distribution across the genome was assessed using bamutils (from ngsutils) (53). Uniquely mapped sequencing reads were assigned to hg38 refGene genes using featureCounts (from subread) (54) with the following parameters: “-s 2 –p –Q 10.” Quality control of sequencing and mapping results were summarized using MultiQC (55). Genes with read count per million <1 in more than four of the samples were removed. The data were normalized using the median of ratios method. Differential expression analysis was performed using DESeq2 (56). Adjusted *p* values were computed from *p* values using the Benjamini–Hochberg procedure. DE genes were determined by log2fold change greater than |1| with an adjusted *p* value less than 0.05. Volcano plots were generated using “EnhancedVolcano” https://github.com/kevinblighe/EnhancedVolcano, and biological pathway analysis of RNA-Seq data was performed using the clusterProfiler package in R (57).

### Splicing analysis

Splicing analysis was performed using rMATS (version 4.1.0) (58). FASTQ files were again aligned with STAR RNA-Seq aligner (version 2.5) to the human genome (UCSC hg38) but with parameters optimized for detection of reads across splice junctions according to the default rMATS settings (52, 58). Specifically the options used were “--chimSegmentMin 2 --outFilterMismatchNmax 3 --alignEndsType EndToEnd --outSAMstrandField intronMotif --alignSJDBoverhangMin 6 --alignIntronMax 299999.” AS events were identified using ILD greater than |0.05| with an FDR less than 0.05.

### KMT assays

Reactions (10 μl) containing 1 μg of KMT, 1 μg of the indicated substrates, and 1 μCi of 3H-SAM (PerkinElmer) in KMT reaction buffer (50 mM Tris [pH 8.8], 5 mM MgCl_2_, and 4 mM DTT) were incubated overnight at room temperature. Reactions were quenched by the addition of SDS loading buffer and resolved by SDS–PAGE. Following the detection of total protein by Coomassie staining, gels were treated with EN3HANCE (PerkinElmer) and dried, and methylated proteins were detected by autoradiography. Nucleosome substrates were purchased from Epicypher (HeLa mononucleosomes [catalog no.: 16-0002]; chicken mononucleosomes [catalog no.: 16-0019]; and recombinant human mononucleosomes [catalog no.: 16-0006]). Isolated histone H3 protein was purchased from Active Motif (catalog no.: 31296). FL recombinant SETMAR was purchased from Active Motif (catalog no.: 31454). G9a/EHMT2 was used as a control KMT. G9a (amino acids 913–1193) was expressed as a HIS-MBP N-terminal fusion in *E. coli* BL21(DE3) and grown in LB media at 37 °C. When the absorbance at 600 nm reached 0.6 to 0.8, the temperature was lowered to 16 °C, IPTG was added (0.5 mM), and incubation was continued overnight with shaking. Bacteria were harvested by centrifugation, and the protein was purified using a HisTrap-HP (Cytiva) followed by size-exclusion chromatography.

## Data availability

The datasets generated or analyzed during the current study are available in the following repositories.

### Crystal structure

The coordinate and data files have been deposited with the RCSB.org, PDB identifier: 7S03.

### RNA-Seq

Data have been deposited with Gene Expression Omnibus (GSE181978).

### ChIP-Seq (SETMAR, HEK293T)

Data have been deposited with Gene Expression Omnibus (GSE103017).

## Supporting information

This article contains supporting information.

## Conflict of interest

R. C. W. has received grant support from Eli Lilly and Company, and R. C. W. serves as a scientific advisor to HiberCell; all other authors declare that they have no conflicts of interest with the contents of this article.

## References

[1] Mills R.E., Bennett E.A., Iskow R.C., Devine S.E. (2007). Which transposable elements are active in the human genome?. Trends Genetics.

[2] Sinzelle L., Izsvak Z., Ivics Z. (2009). Molecular domestication of transposable elements: From detrimental parasites to useful host genes. Cell Mol. Life Sci..

[3] Pace J.K., Feschotte C. (2007). The evolutionary history of human DNA transposons: Evidence for intense activity in the primate lineage. Genome Res..

[4] Robertson H.M., Zumpano K.L. (1997). Molecular evolution of an ancient mariner transposon, Hsmar1, in the human genome. Gene.

[5] Cordaux R., Udit S., Batzer M.A., Feschotte C. (2006). Birth of a chimeric primate gene by capture of the transposase gene from a mobile element. Proc. Natl. Acad. Sci. U. S. A..

[6] Tellier M., Chalmers R. (2019). Human SETMAR is a DNA sequence-specific histone-methylase with a broad effect on the transcriptome. Nucleic Acids Res..

[7] Antoine-Lorquin A., Arensburger P., Arnaoty A., Asgari S., Batailler M., Beauclair L., Belleannee C., Buisine N., Coustham V., Guyetant S., Helou L., Lecomte T., Pitard B., Stevant I., Bigot Y. (2021). Two repeated motifs enriched within some enhancers and origins of replication are bound by SETMAR isoforms in human colon cells. Genomics.

[8] Uhlen M., Fagerberg L., Hallstrom B.M., Lindskog C., Oksvold P., Mardinoglu A., Sivertsson A., Kampf C., Sjostedt E., Asplund A., Olsson I., Edlund K., Lundberg E., Navani S., Szigyarto C.A. (2015). Proteomics. Tissue-based map of the human proteome. Science.

[9] Liu D., Bischerour J., Siddique A., Buisine N., Bigot Y., Chalmers R. (2007). The human SETMAR protein preserves most of the activities of the ancestral Hsmar1 transposase. Mol. Cell Biol..

[10] Roman Y., Oshige M., Lee Y.J., Goodwin K., Georgiadis M.M., Hromas R.A., Lee S.H. (2007). Biochemical characterization of a SET and transposase fusion protein, Metnase: Its DNA binding and DNA cleavage activity. Biochemistry.

[11] Miskey C., Papp B., Mates L., Sinzelle L., Keller H., Izsvak Z., Ivics Z. (2007). The ancient mariner sails again: Transposition of the human Hsmar1 element by a reconstructed transposase and activities of the SETMAR protein on transposon ends. Mol. Cell Biol..

[12] Lee S.H., Oshige M., Durant S.T., Rasila K.K., Williamson E.A., Ramsey H., Kwan L., Nickoloff J.A., Hromas R. (2005). The SET domain protein Metnase mediates foreign DNA integration and links integration to nonhomologous end-joining repair. Proc. Natl. Acad. Sci. U. S. A..

[13] Wray J., Williamson E.A., Sheema S., Lee S.H., Libby E., Willman C.L., Nickoloff J.A., Hromas R. (2009). Metnase mediates chromosome decatenation in acute leukemia cells. Blood.

[14] De Haro L.P., Wray J., Williamson E.A., Durant S.T., Corwin L., Gentry A.C., Osheroff N., Lee S.H., Hromas R., Nickoloff J.A. (2010). Metnase promotes restart and repair of stalled and collapsed replication forks. Nucleic Acids Res..

[15] Tellier M., Chalmers R. (2019). The roles of the human SETMAR (Metnase) protein in illegitimate DNA recombination and non-homologous end joining repair. DNA Repair (Amst).

[16] Carlson S.M., Moore K.E., Sankaran S.M., Elias J.E., Gozani O. (2015). A proteomic strategy identifies lysine methylation of splicing factor snRNP70 by SETMAR. J. Biol. Chem..

[17] Baralle F.E., Giudice J. (2017). Alternative splicing as a regulator of development and tissue identity. Nat. Rev. Mol. Cell Biol..

[18] Goodwin K.D., He H., Imasaki T., Lee S.H., Georgiadis M.M. (2010). Crystal structure of the human Hsmar1-derived transposase domain in the DNA repair enzyme Metnase. Biochemistry.

[19] Chen Q., Georgiadis M. (2016). Crystallization of and selenomethionine phasing strategy for a SETMAR-DNA complex. Acta Crystallogr. Section F, Struct. Biol. Commun..

[20] Meng E.C., Pettersen E.F., Couch G.S., Huang C.C., Ferrin T.E. (2006). Tools for integrated sequence-structure analysis with UCSF Chimera. BMC Bioinformatics.

[21] Droit A., Gottardo R., Robertson G., Li L. (2014).

[22] Miskei M., Horvath A., Viola L., Varga L., Nagy E., Fero O., Karanyi Z., Roszik J., Miskey C., Ivics Z., Szekvolgyi L. (2021). Genome-wide mapping of binding sites of the transposase-derived SETMAR protein in the human genome. Comput. Struct. Biotechnol. J..

[23] Koudritsky M., Domany E. (2008). Positional distribution of human transcription factor binding sites. Nucleic Acids Res..

[24] Perelman P., Johnson W.E., Roos C., Seuanez H.N., Horvath J.E., Moreira M.A., Kessing B., Pontius J., Roelke M., Rumpler Y., Schneider M.P., Silva A., O'Brien S.J., Pecon-Slattery J. (2011). A molecular phylogeny of living primates. PLoS Genet..

[25] Jeyaratnam D.C., Baduin B.S., Hansen M.C., Hansen M., Jorgensen J.M., Aggerholm A., Ommen H.B., Hokland P., Nyvold C.G. (2014). Delineation of known and new transcript variants of the SETMAR (Metnase) gene and the expression profile in hematologic neoplasms. Exp. Hematol..

[26] Dussaussois-Montagne A., Jaillet J., Babin L., Verrelle P., Karayan-Tapon L., Renault S., Rousselot-Denis C., Zemmoura I., Auge-Gouillou C. (2017). SETMAR isoforms in glioblastoma: A matter of protein stability. Oncotarget.

[27] Ran F.A., Hsu P.D., Lin C.Y., Gootenberg J.S., Konermann S., Trevino A.E., Scott D.A., Inoue A., Matoba S., Zhang Y., Zhang F. (2013). Double nicking by RNA-guided CRISPR Cas9 for enhanced genome editing specificity. Cell.

[28] Ran F.A., Hsu P.D., Wright J., Agarwala V., Scott D.A., Zhang F. (2013). Genome engineering using the CRISPR-Cas9 system. Nat. Protoc..

[29] Cornett E.M., Ferry L., Defossez P.A., Rothbart S.B. (2019). Lysine methylation regulators moonlighting outside the epigenome. Mol. Cell.

[30] Ruiz-Velasco M., Kumar M., Lai M.C., Bhat P., Solis-Pinson A.B., Reyes A., Kleinsorg S., Noh K.M., Gibson T.J., Zaugg J.B. (2017). CTCF-mediated chromatin loops between promoter and gene body regulate alternative splicing across individuals. Cell Syst..

[31] Shukla S., Kavak E., Gregory M., Imashimizu M., Shutinoski B., Kashlev M., Oberdoerffer P., Sandberg R., Oberdoerffer S. (2011). CTCF-promoted RNA polymerase II pausing links DNA methylation to splicing. Nature.

[32] Achsel T., Brahms H., Kastner B., Bachi A., Wilm M., Luhrmann R. (1999). A doughnut-shaped heteromer of human Sm-like proteins binds to the 3'-end of U6 snRNA, thereby facilitating U4/U6 duplex formation *in vitro*. EMBO J..

[33] Bertram K., Agafonov D.E., Dybkov O., Haselbach D., Leelaram M.N., Will C.L., Urlaub H., Kastner B., Luhrmann R., Stark H. (2017). Cryo-EM structure of a pre-catalytic human spliceosome primed for activation. Cell.

[34] Gaudet P., Livstone M.S., Lewis S.E., Thomas P.D. (2011). Phylogenetic-based propagation of functional annotations within the Gene Ontology consortium. Brief Bioinform..

[35] Liu J., Liu W., Yang L., Wu Q., Zhang H., Fang A., Li L., Xu X., Sun L., Zhang J., Tang F., Wang X. (2017). The primate-specific gene TMEM14B marks outer radial glia cells and promotes cortical expansion and folding. Cell Stem Cell.

[36] Carlson S.M., Gozani O. (2014). Emerging technologies to map the protein methylome. J. Mol. Biol..

[37] Sinha K.M., Yasuda H., Coombes M.M., Dent S.Y., de Crombrugghe B. (2010). Regulation of the osteoblast-specific transcription factor Osterix by NO66, a Jumonji family histone demethylase. EMBO J..

[38] Oh S., Shin S., Janknecht R. (2019). The small members of the JMJD protein family: Enzymatic jewels or jinxes?. Biochim. Biophys. Acta Rev. Cancer.

[39] Kuo A.J., Cheung P., Chen K., Zee B.M., Kioi M., Lauring J., Xi Y., Park B.H., Shi X., Garcia B.A., Li W., Gozani O. (2011). NSD2 links dimethylation of histone H3 at lysine 36 to oncogenic programming. Mol. Cell.

[40] Kowalski M.P., Krude T. (2015). Functional roles of non-coding Y RNAs. Int. J. Biochem. Cell Biol..

[41] Kim H.S., Chen Q., Kim S.K., Nickoloff J.A., Hromas R., Georgiadis M.M., Lee S.H. (2014). The DDN catalytic motif is required for Metnase functions in non-homologous end joining (NHEJ) repair and replication restart. J. Biol. Chem..

[42] Kabsch W. (2010). Xds. Acta Crystallogr. Sect. D, Biol. Crystallogr..

[43] Minor W., Cymborowski M., Otwinowski Z., Chruszcz M. (2006). HKL-3000: The integration of data reduction and structure solution--from diffraction images to an initial model in minutes. Acta Crystallogr. Sect. D, Biol. Crystallogr..

[44] Adams P.D., Afonine P.V., Bunkoczi G., Chen V.B., Davis I.W., Echols N., Headd J.J., Hung L.W., Kapral G.J., Grosse-Kunstleve R.W., McCoy A.J., Moriarty N.W., Oeffner R., Read R.J., Richardson D.C. (2010). Phenix: A comprehensive python-based system for macromolecular structure solution. Acta Crystallogr. Section D, Biol. Crystallogr..

[45] Emsley P., Lohkamp B., Scott W.G., Cowtan K. (2010). Features and development of coot. Acta Crystallogr. Sect. D, Biol. Crystallogr..

[46] McCoy A.J., Grosse-Kunstleve R.W., Adams P.D., Winn M.D., Storoni L.C., Read R.J. (2007). Phaser crystallographic software. J. Appl. Crystallogr..

[47] Bricogne G.B.E., Brandl M., Flensburg C., Keller P., Paciorek W., Roversi P.S.A., Smart O.S., Vonrhein C., Womack T.O. (2016).

[48] Li L. (2009). Gadem: A genetic algorithm guided formation of spaced dyads coupled with an EM algorithm for motif discovery. J. Comput. Biol..

[49] Lawrence M., Huber W., Pages H., Aboyoun P., Carlson M., Gentleman R., Morgan M.T., Carey V.J. (2013). Software for computing and annotating genomic ranges. PLoS Comput. Biol..

[50] Grant C.E., Bailey T.L., Noble W.S. (2011). FIMO: Scanning for occurrences of a given motif. Bioinformatics.

[51] Korhonen J., Martinmaki P., Pizzi C., Rastas P., Ukkonen E. (2009). MOODS: Fast search for position weight matrix matches in DNA sequences. Bioinformatics.

[52] Dobin A., Davis C.A., Schlesinger F., Drenkow J., Zaleski C., Jha S., Batut P., Chaisson M., Gingeras T.R. (2013). STAR: Ultrafast universal RNA-seq aligner. Bioinformatics.

[53] Breese M.R., Liu Y. (2013). NGSUtils: A software suite for analyzing and manipulating next-generation sequencing datasets. Bioinformatics.

[54] Liao Y., Smyth G.K., Shi W. (2014). featureCounts: An efficient general purpose program for assigning sequence reads to genomic features. Bioinformatics.

[55] Ewels P., Magnusson M., Lundin S., Kaller M. (2016). MultiQC: Summarize analysis results for multiple tools and samples in a single report. Bioinformatics.

[56] Love M.I., Huber W., Anders S. (2014). Moderated estimation of fold change and dispersion for RNA-seq data with DESeq2. Genome Biol..

[57] Yu G., Wang L.G., Han Y., He Q.Y. (2012). clusterProfiler: An R package for comparing biological themes among gene clusters. OMICS.

[58] Shen S., Park J.W., Lu Z.X., Lin L., Henry M.D., Wu Y.N., Zhou Q., Xing Y. (2014). rMATS: Robust and flexible detection of differential alternative splicing from replicate RNA-Seq data. Proc. Natl. Acad. Sci. U. S. A..

[59] Huang W., Loganantharaj R., Schroeder B., Fargo D., Li L. (2013). Pavis: A tool for peak annotation and visualization. Bioinformatics.

