# Supplementary material for: Structural and genome-wide analyses suggest that transposon-derived protein SETMAR alters transcription and splicing
Source: J Biol Chem. 2022 Apr 1;298(5):101894. doi: 10.1016/j.jbc.2022.101894 (PMC9062482; doi:10.1016/j.jbc.2022.101894)
Supplement: Supplemental Table S1 and Figures S1–S10 [file mmc1.docx]

**Supporting Information**

**SETMAR regulates gene expression and alternative splicing**

Qiujia Chen,^1#^ Alison M. Bates,^1#^ Jocelyne N. Hanquier,^2^ Edward Simpson,^3^ Douglas B. Rusch,^4^ Ram Podicheti,^4^ Yunlong Liu,^3^ Ronald C. Wek,^1^ Evan M. Cornett,^1,2^ and Millie M. Georgiadis^1^*

^1^Department of Biochemistry and Molecular Biology, ^2^ Stark Neurosciences Research Institute, ^3^Department of Medical and Molecular Genetics, Indiana University School of Medicine, Indianapolis, IN 46202, USA; ^4^Center for Genomics and Bioinformatics, Indiana University, Bloomington, IN 47405

# These authors contributed equally to the manuscript.

* To whom correspondence should be addressed. Tel: +1 317 278 8486; Fax: +1 317 274 4686; Email: [mgeorgia@iu.edu](mailto:mgeorgia@iu.edu)

| Table S1. Crystallographic Data | | |
| --- | --- | --- |
|  | **TIR complex (Se-SAD)** | **TIR complex (High Res.)** |
| PDB ID |  | 6XMZ |
| Data Collection |  |  |
| Space group | C222_1_ | C222_1_ |
| Cell dimensions |  |  |
| a, b, c (Å) | 70.09, 166.04, 66.09 | 70.98, 166.17, 66.05 |
| α, β, γ (°) | 90, 90, 90 | 90, 90, 90 |
| Wavelength (Å) | 0.97938 | 0.97938 |
| Resolution (Å) | 29.42—2.66 | 27.70—2.37 (2.46—2.37) |
| *R*_merge_ | 0.056 (0.422) | 0.028 (0.393) |
| *R*_pim_ | 0.027 | 0.024 (0.256) |
| CC(1/2) | 0.990 | 0.999 |
| Mean I/σ(I) | 18.4 | 21.9 (2.3) |
| Completeness (%) | 99.5 | 99.7 (99.1) |
| Redundancy | 7.7 | 4.3 (4.0) |
| Refinement |  |  |
| Resolution (Å) | - | 27.70---2.37 |
| No. reflections | - | 16176 |
| *R*_work /_ *R*_free_ (%) | - | 21.10 / 23.50 |
| No. atoms | - |  |
| Protein | - | 884 |
| DNA | - | 1060 |
| Water | - | 13 |
| *B* factors | - |  |
| Protein | - | 73.23 |
| DNA | - | 89.69 |
| Water | - | 71.07 |
| r.m.s. deviation | - |  |
| Bond lengths (Å) | - | 0.013 |
| Bond angles (°) | - | 1.34 |


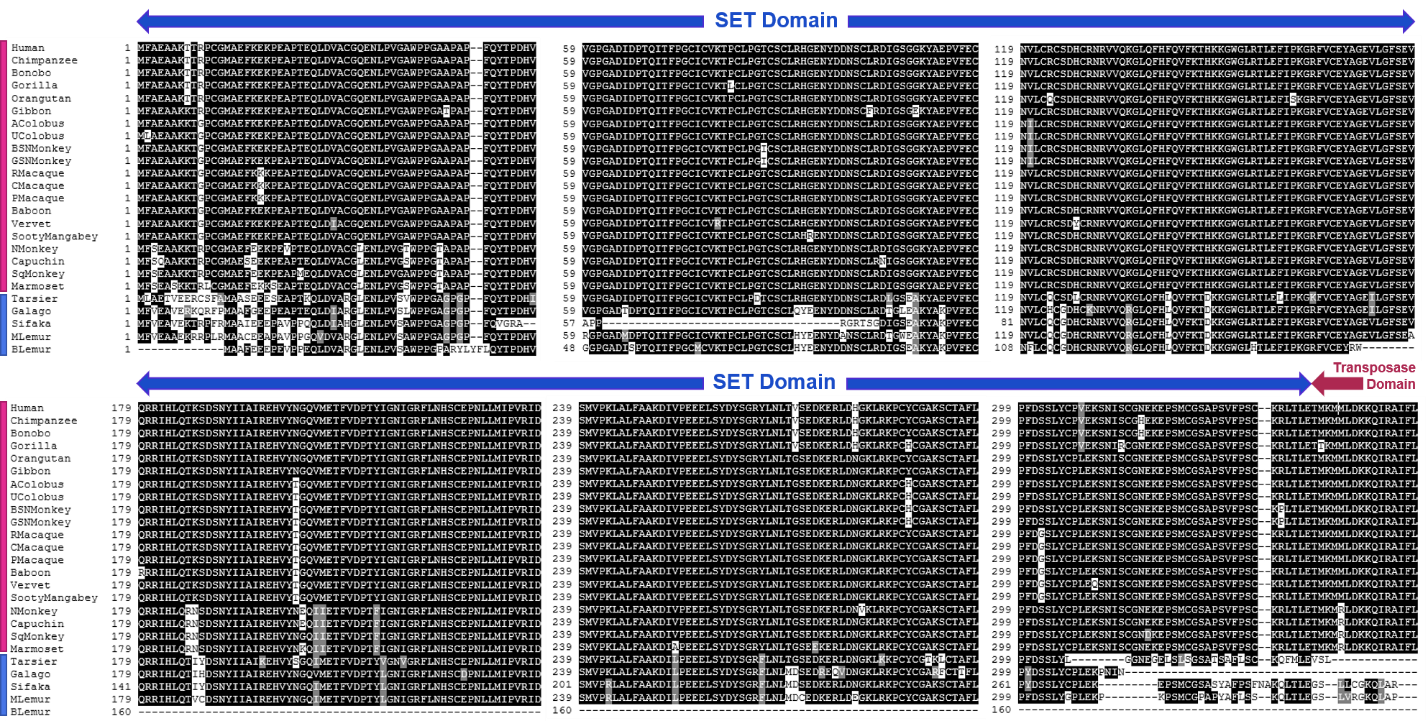


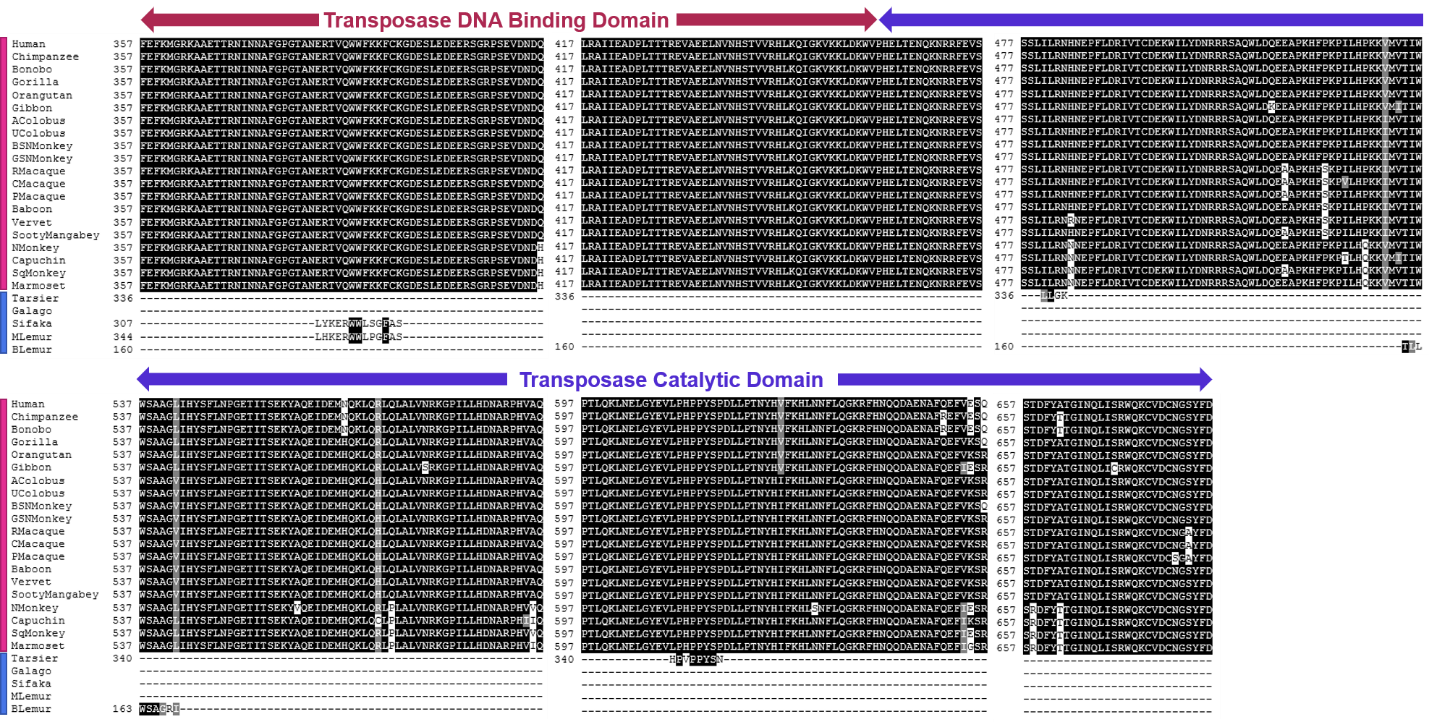


**SI Figure S1**. Alignment of SETMAR gene sequences in selected primates. The sequences of the SETMAR gene in 19 anthropoid primates and 5 non-anthropoid primates for which whole genome sequences are available were aligned with the human sequence. The highest degree of sequence conservation occurs in the DNA-binding portion of the mariner transposase domain.

**
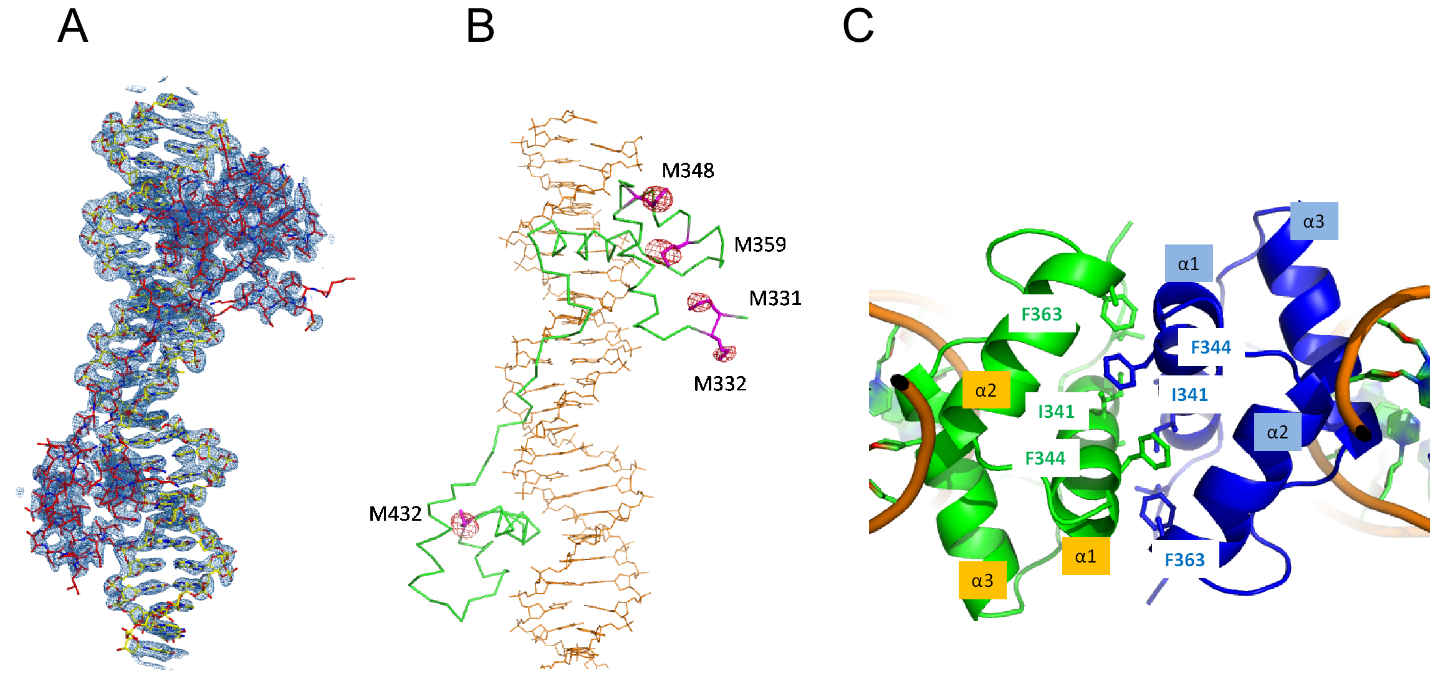
**

**SI Figure S2.** (A) The 2.66 Å experimental electron density map of TIR complex, from selenium single wavelength anomalous diffraction (Se-SAD) phasing (blue mesh, contoured at 1.6 sigma) is shown superimposed on the refined model. The protein backbone atoms are shown in red, and the DNA backbone atoms are shown in yellow. The crystal structure includes a single DNA-binding domain of SETMAR bound to a TIR-containing oligonucleotide in the unique, repeating unit of the crystal (asymmetric unit). The dimeric structure is created by crystallographic symmetry in the crystal. (B) Anomalous difference Fourier map of the SeMet-labeled TIR complex, superimposed with the backbone trace of the refined model (protein, green and DNA, orange stick model). The map is contoured at 4 sigma (red). (SeMet 331, 332, 348, 359, and 423 are displayed as ball-and-stick side chain models (magenta). (C) The DBD dimerizes through protein-protein interactions of its HTH1 motifs. Hydrophobic residues involved in the interface are shown as sticks. Three representative residues of the hydrophobic cluster, F363, F344, and I341 are labeled.

**
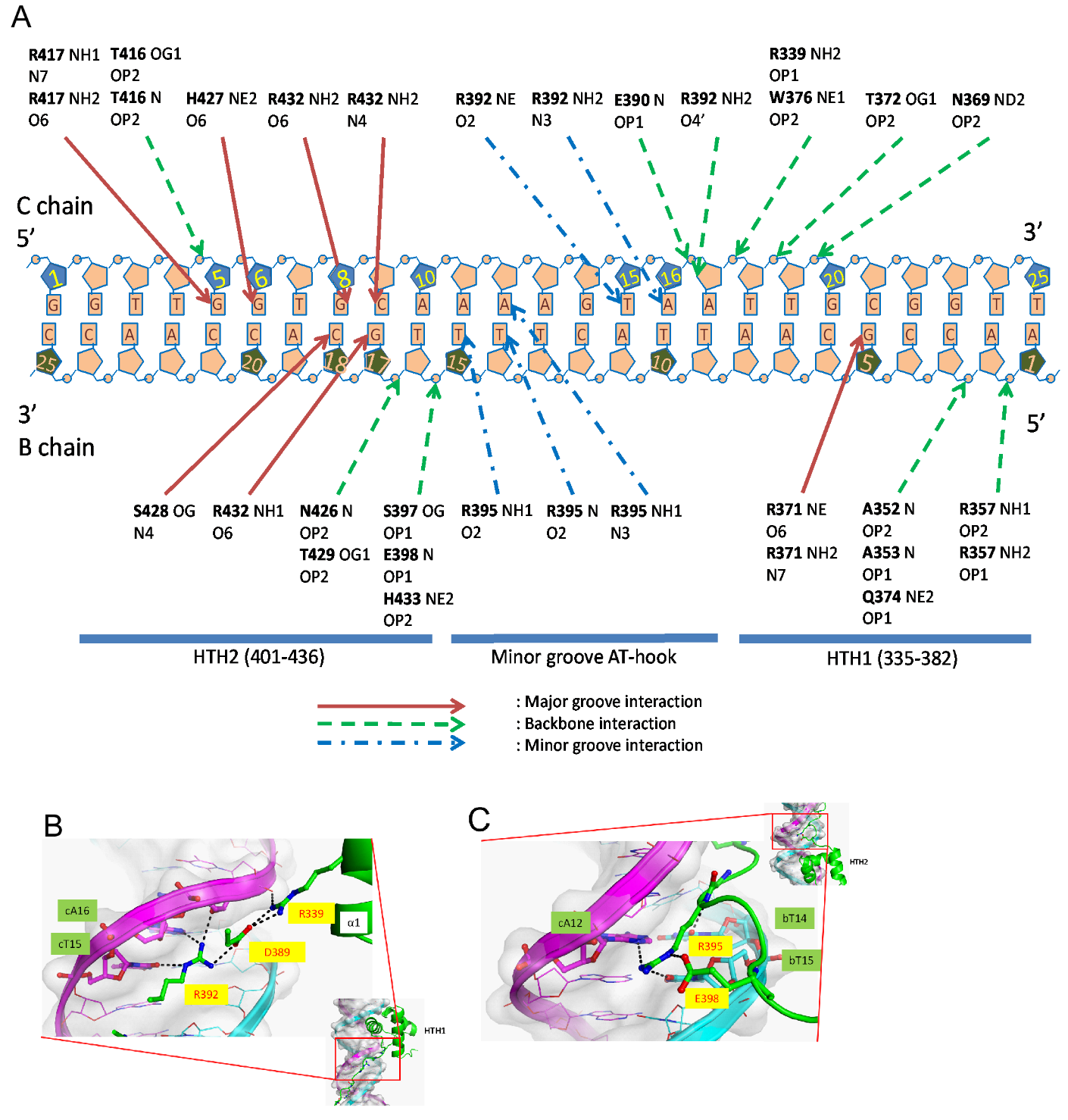
**

**SI Figure S3.** SETMAR interacts extensively with TIR DNA. (A) Red arrows indicate major groove base-specific contacts, blue dotted arrows show minor groove contacts, and green broken arrows represent interactions between the protein and DNA backbone. (B) The interface between the N-terminal linker region and the DNA minor groove. The protein is rendered as a ribbon diagram with DNA as a line and surface rendering. Oxygen and nitrogen atoms are shown in red and blue, respectively. Key residue side chains and bases are shown as ball-and-stick models. Hydrogen bonds are shown in black broken lines. Arg-339 and Asp-389 orient Arg-392 to make contacts with DNA bases in the minor groove. (C) The interface between the C-terminal linker region and the DNA minor groove. In a classic bifurcated type of interaction mode, NH1 of Arg-395 hydrogen bonds with two bases: A12 at chain C (labeled as cA12) and T15 at chain B (labeled as bT15). OE2 of Glu-398 makes a hydrogen bond with NE atom of Arg-395.

**

**

**SI Figure S4.** The binding affinity of full-length SETMAR for a TIR-containing DNA. Rhodamine-labeled TIR (20 nM) was titrated with increasing amounts of SETMAR protein. Binding curves were fitted for protein concentration versus FA signal. A K_D_ value of 53 ± 4 nM was calculated for TIR binding. The results are reported for triplicate measurements done for three independent assays.


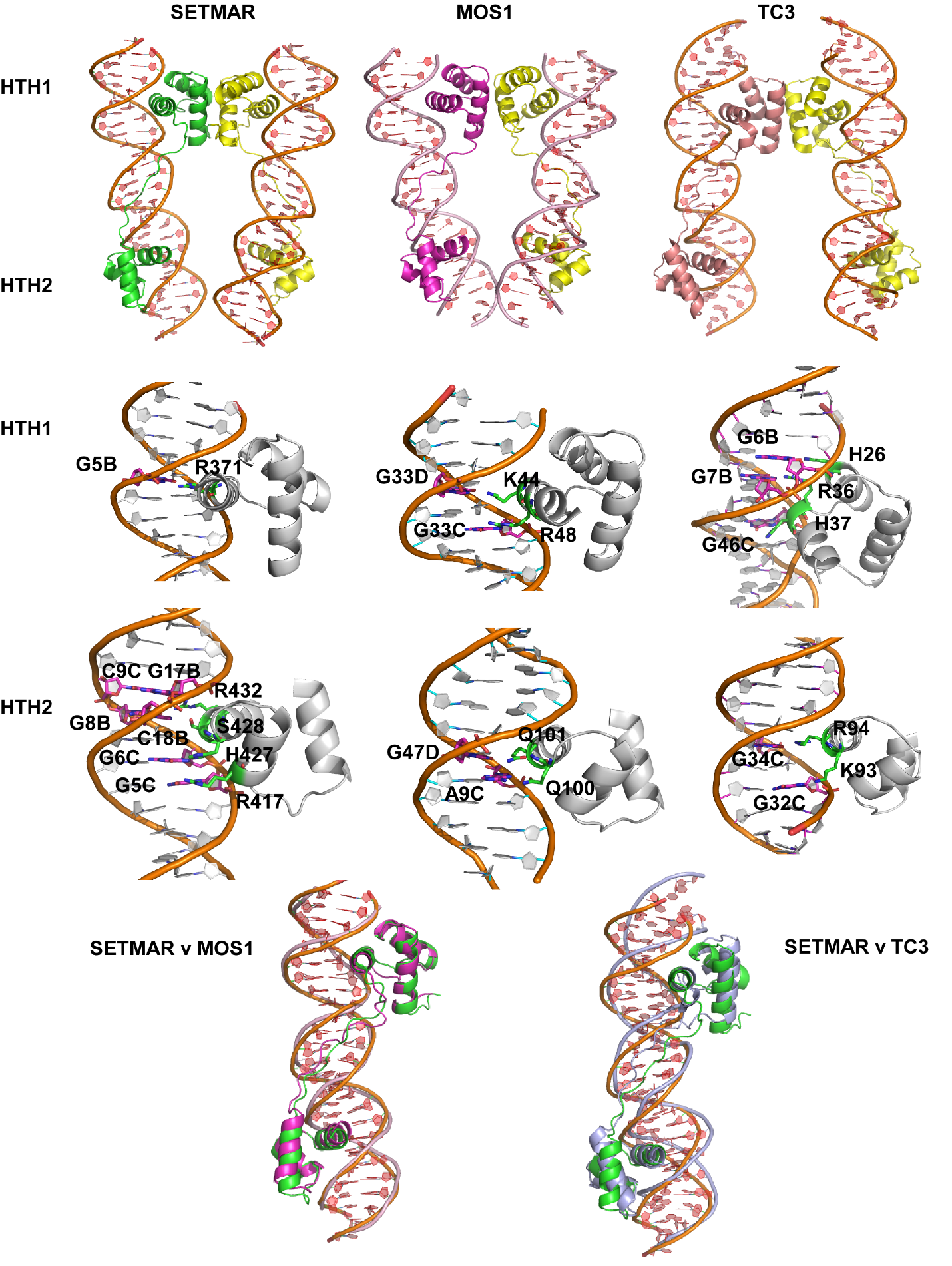


**SI Fig. S5.** Comparison of SETMAR with related transposase structures. SETMAR (this work), MOS1 (3HOT), and TC3 (U178) are shown. Each of these structures includes two DNA-binding domains shown as cartoon renderings in green and yellow for SETMAR, magenta and yellow for MOS1, and salmon and yellow for TC3 with bound DNA as a cartoon rendering. Each DNA-binding domain contains two HTH motifs, HTH1 and HTH2 connected by a linker. Both SETMAR and TC3 contain a single DNA-binding domain and bound DNA molecule in the asymmetric unit; the dimer is generated by crystallographic symmetry. The structure of MOS1 includes the full-length transposase in a paired end complex. Shown here are the two DNA-binding domains with bound cognate DNA molecules from the structure (3HOT). In each case, the protein dimerizes through interactions between alpha helix 1 in HTH1. Close-up views of nucleobase sequence-specific interactions with amino acids (green stick renderings) in HTH1 and HTH2 and nucleobases (magenta stick renderings) are shown below each structure, each HTH domain is shown as a cartoon rendering in gray. For HTH1 motifs, there is one residue that makes a nucleobase-specific interaction in SETMAR, two residues in MOS1, and 3 residues in TC3. For HTH2 motifs, there are 4 residues that make nucleobase-specific interactions in SETMAR, and two in MOS1 and TC3. In the bottom panel, the SETMAR DBD (green) with bound DNA is shown superimposed with MOS1 (magenta) or TC3 (pale blue). The structures were aligned based on the DBDs in Matchmaker implemented in Chimera(1). Matchmaker excludes residues with matches greater than 2 Å to superimpose the molecules and also provides the rmsd for all Cα that are structurally equivalent. The rmsd for alignment of 106 Cα atoms in SETMAR and MOS1 is 1.9 Å and for alignment of 96 Cα atoms in SETMAR and TC3 is 4.9 Å. While HTH1 motifs in both SETMAR and MOS1 are close in size and larger than HTH2 motifs, the HTH1 motif in TC3 is smaller.

**Structure-based sequence alignment of SETMAR, MOS1, and TC3**

330 K....MMLD......KK.QIRAIFLFE.FKMG.RKA..A.ETTRNMNNAF 363 SETMAR

5 .MSSFV.PN......KE.QTRTVLIFC.FHLK.KTA..A.ESHRMLVEAF 36 MOS1

1 .........MPRGSALSDTERAQL.DVMK.L.LN..VSLHEMSR..K... 31 TC3

364 GPG.TANERTVQWWFKKFRKGD.ESL.ED..EER..SGRPSEV.D..NDQ 403 SETMAR

37 GEQ.VPTVKTCERWFQRFKSG..DFDVDD..K.E.HGKPPKRY.E..DAE 76 MOS1

32 ...ISRSRHCIRVYLK.D....PVSY..GTSK.RA.P.RRKALSVRDERN 68 TC3

404 LRAIIE.ADPLTTTREVAE.E.MNVNHSTVVRHLKQI. 437 SETMAR

77 LQALLDEDDA.QTQKQLAE.Q.LEVSQQAVSNRLREMG 111 MOS1

69 VIRAAS..NSCKTARDIRNELQLSASKRTILNVIKRSG 104 TC3

**Structural alignment of DNA sequences**

C 5’ GGTTGGTGCAAAAGTAATTGCGGTTA SETMAR

B 3’ TCCAACCACGTTTTCATTAACGCCAA

C 5’ GGTGTACAAGTATGAAATGTCGTTT MOS1

D 3’ AGTCCACATGTTCATACTTTACAGCAAA

C 3’ TGTGGGAAAGTTCTATAGGACCCCCG TC3

B 5’ CACACCCTTTCAAGATATCCTGGGGG

**SI Fig. S6**. In the top panel, a structure-based alignment for the amino acid sequences is shown for SETMAR, MOS1, and TC3 DNA-binding domains. This alignment was produced using the Match->align tool in Chimera following superpositioning by Matchmaker. Sequence identity is 25% for SETMAR vs MOS1 and 9% for SETMAR vs TC3. Residues that make nucleobase-specific contacts in each protein are highlighted in green for HTH1 and yellow for HTH2 with structurally equivalent residues involved in nucleobase specific recognition underlined. While there is conservation of the position in HTH1 equivalent to R371 in SETMAR, this residue is different in each structure, K44 in MOS1 and H37 in TC3. The number of residues that make nucleobase specific contacts in both HTH1 and HTH2 are also different with one in SETMAR, two in MOS1, and three in TC3. Within HTH2, two residues that make nucleobase specific contacts in each structure are structurally equivalent, S427/H428 in SETMAR, Q100/Q101 in MOS1, and K93/R94 in TC3. The residues are different in each structure and the total number of residues that make nucleobase-specific contacts are different with four in SETMAR, two in MOS1, and two in TC3. In the bottom panel, the DNA sequences in each structure have been aligned based on alignment of the protein structures in Matchmaker (Chimera). MOS1 exhibits the smallest number of nucleobase-specific interactions with 4, SETMAR the most with 7; TC3 has 5. R392 and R395 (highlighted in cyan) within AT hook elements are conserved in TC3 but not MOS1. While most of the nucleobase-specific contacts involve G, most of these G nucleobases are not all structurally equivalent in the structures. Structurally equivalent Gs involved in nucleobase specific recognition by the protein are underlined. There is only one conserved G in both SETMAR and MOS1 interactions and two in SETMAR and TC3 interactions. Thus, even though the structures exhibit similar folds, they form unique complexes with respective DNA recognition elements. As a consequence, none of the structurally equivalent amino acid residues involved in sequence specific DNA recognition are identical in the three structures.

**A.**

**
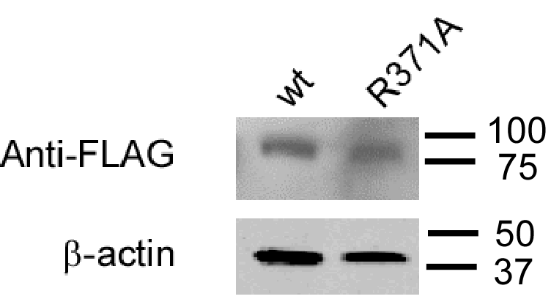
**

**B.**

**C.**


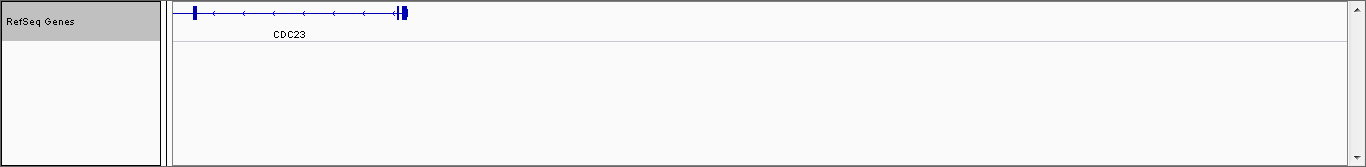

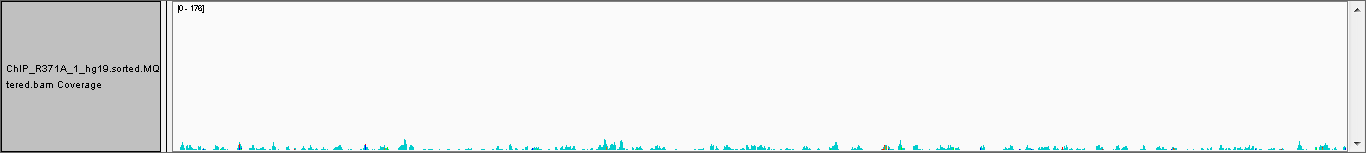

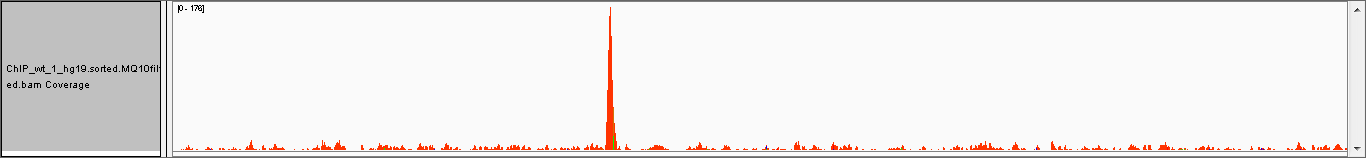


**SI Fig. S7. (A)** Western-blot of FLAG-tagged SETMAR (wt) and FLAG-tagged SETMAR (R371A), showing that amino acid substitution (R371A) does not affect the protein expression level or the stability in this transient overexpression experiment. Molecular weight markers (kDa) are indicated on the right of the image. **(B)** Quantitative PCR was performed to validate the ChIP experiment for binding of SETMAR to a perfect TIR site found upstream of CDC23 (N=3, p, 0.0001). (**C)** Image of ChIP-seq reads from IGV for wild-type SETMAR (red) and R371A SETMAR (cyan) samples.

**
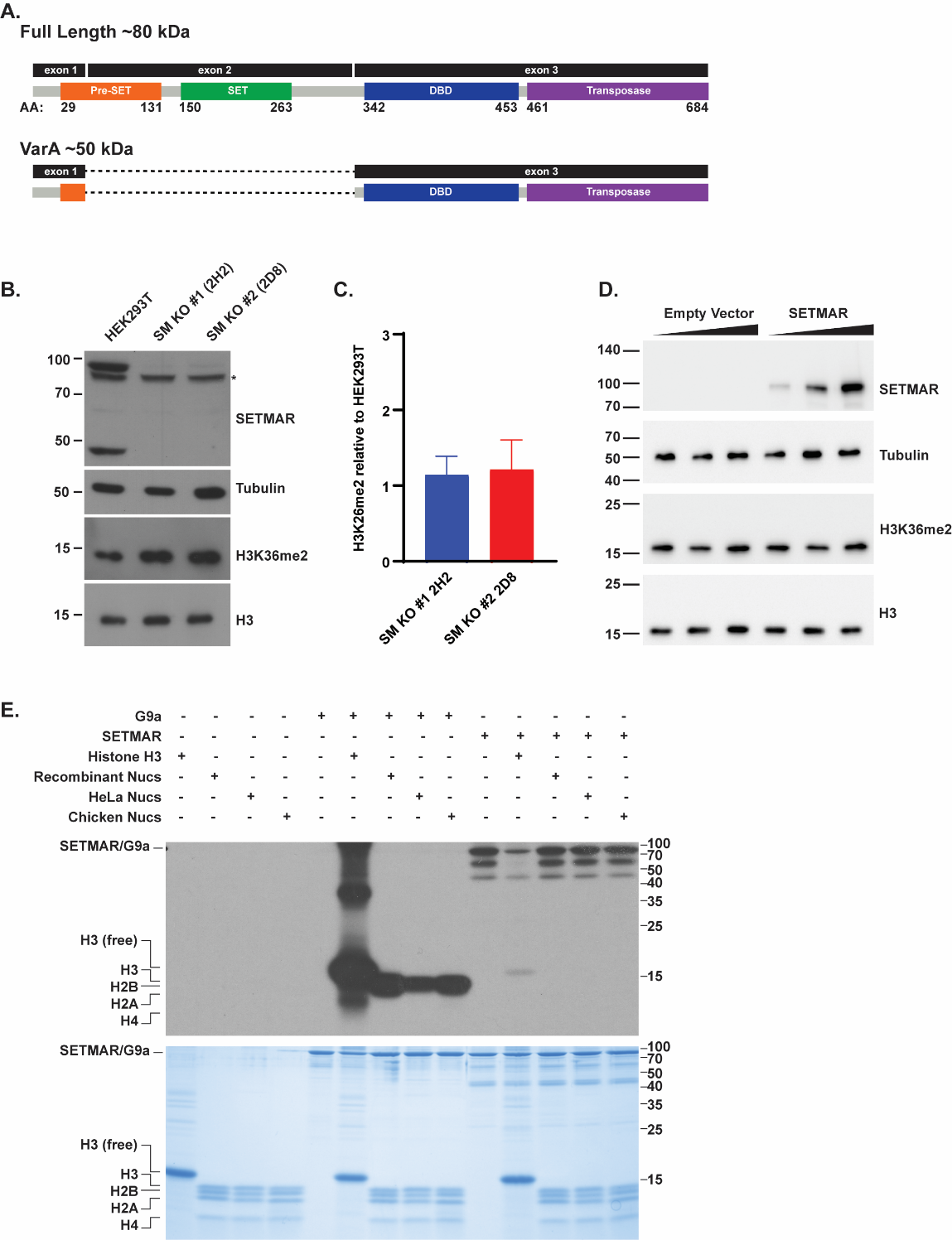
**

**SI Fig. S8.** (A) Domain architecture of SETMAR isoforms. Domain boundaries are derived from structural information with DBD referring to the DNA-binding domain and transposase, the catalytic domain of the mariner tranposase. (B) Western blot analysis of SETMAR KO clones. Full-length SETMAR (FL) and the shorter splice variant lacking the SET domain (VarA) are indicated. A non-specific band present in all samples is denoted with an asterisk. (C) H3K36me2 signal from three independent experiments was normalized to H3 as a loading control and quantified using ImageJ. The bar chart shows the mean normalized intensity relative to the parental cells. Error bars represent the standard deviation. (D) Western blot analysis of HEK293T lysates with overexpression of full-length SETMAR. (E) *In vitro* lysine methyltransferase assays. Top shows autoradiography after exposing dried, ENHANCE treated gels to film for 2 days. The bottom panel shows the corresponding Coomassie blue-stained gel image.


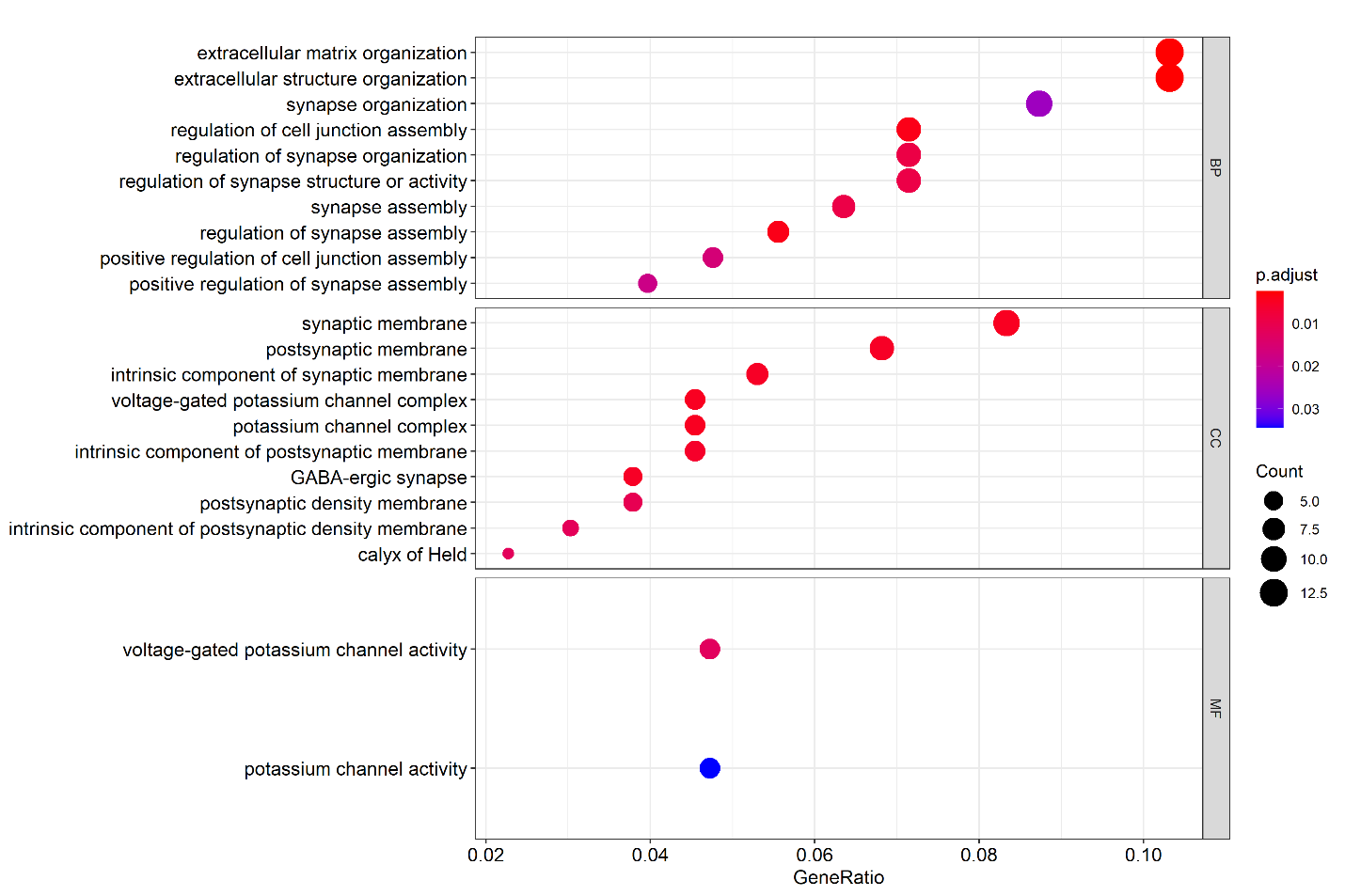


**SI Fig. S9.** Over-representation analysis of the 163 common differentially expressed genes with expression in the same direction in SETMAR KO#1 and KO#2 using the enrichGO function from the “clusterProfiler” package in R. Enriched ontologies- biological processes (“BP”), cellular compartments (“CC”), and molecular functions (“MF”)- identified using p. adjusted method of FDR with a cutoff of 0.05.

**SI Fig. S10.** Overlap between TIRs and enhancer elements. SI Fig. Enhancer information was downloaded from the Fantom5 database: <https://slidebase.binf.ku.dk/human_enhancers/bed> and from the Enhancer Atlas: <http://enhanceratlas.org/data/download/enhancer/hs/HEK293T.bed>. The x-axis value of 0 refers to the start of the SETMAR binding site in the + orientation. The negative x-axis values are upstream (5’) of the binding site while the positive x-axis values are downstream (3’) of the first base of the SETMAR binding site. The y-axis shows the number of enhancer elements that were found at the given distance. Only SETMAR elements with 2 or fewer mismatches were used. The enhancer elements were derived from the FANTOM5 (orange) database or from the Enhancer Atlas (grey; hek293t cell-line)[1]. A total of 1 million sites were randomly chosen across the length of the human genome and analyzed as if they were SETMAR elements. After normalizing the data to the number of known SETMAR sites, this random sites was plotted relative to the FANTOM5 (blue) database or the Enhancer Atlas (gold). The data was generated using the bedtools software suite using the “intersect” algorithm with the “-wb” option. The number of intersections between the Fantom5 and Enhancer Atlas with the SETMAR binding sites was 236 and 146, respectively, out of 5,253 SETMAR selected sites.

**References**

1. Meng, E. C., Pettersen, E. F., Couch, G. S., Huang, C. C., and Ferrin, T. E. (2006) Tools for integrated sequence-structure analysis with UCSF Chimera. *BMC Bioinformatics* **7**, 339
